# Novel Genes Associated with Colorectal Cancer Are Revealed by High Resolution Cytogenetic Analysis in a Patient Specific Manner

**DOI:** 10.1371/journal.pone.0076251

**Published:** 2013-10-30

**Authors:** Hisham Eldai, Sathish Periyasamy, Saeed Al Qarni, Maha Al Rodayyan, Sabeena Muhammed Mustafa, Ahmad Deeb, Ebthehal Al Sheikh, Mohammed Afzal Khan, Mishal Johani, Zeyad Yousef, Mohammad Azhar Aziz

**Affiliations:** 1 Bioinformatics, King Abdullah International Medical Research Center, Riyadh, Saudi Arabia; 2 Medical Biotechnology, King Abdullah International Medical Research Center, Riyadh, Saudi Arabia; 3 Research Office, King Abdullah International Medical Research Center, Riyadh, Saudi Arabia; 4 Anatomic pathology, National Guard Health Affairs, Riyadh, Saudi Arabia; 5 Endoscopy, National Guard Health Affairs, Riyadh, Saudi Arabia; 6 Surgery, National Guard Health Affairs, Riyadh, Saudi Arabia; Deutsches Krebsforschungszentrum, Germany

## Abstract

Genomic abnormalities leading to colorectal cancer (CRC) include somatic events causing copy number aberrations (CNAs) as well as copy neutral manifestations such as loss of heterozygosity (LOH) and uniparental disomy (UPD). We studied the causal effect of these events by analyzing high resolution cytogenetic microarray data of 15 tumor-normal paired samples. We detected 144 genes affected by CNAs. A subset of 91 genes are known to be CRC related yet high GISTIC scores indicate 24 genes on chromosomes 7, 8, 18 and 20 to be strongly relevant. Combining GISTIC ranking with functional analyses and degree of loss/gain we identify three genes in regions of significant loss (ATP8B1, NARS, and ATP5A1) and eight in regions of gain (CTCFL, SPO11, ZNF217, PLEKHA8, HOXA3, GPNMB, IGF2BP3 and PCAT1) as novel in their association with CRC. Pathway and target prediction analysis of CNA affected genes and microRNAs, respectively indicates TGF-β signaling pathway to be involved in causing CRC. Finally, LOH and UPD collectively affected nine cancer related genes. Transcription factor binding sites on regions of >35% copy number loss/gain influenced 16 CRC genes. Our analysis shows patient specific CRC manifestations at the genomic level and that these different events affect individual CRC patients differently.

## Introduction

Colorectal cancer is one of the most prevalent cancers and is a worldwide healthcare concern [1]. It is the most common type of cancer in Asia [2]. The incidence of CRC is increasing among the local population of Saudi Arabia as seen by over a three-fold incidence rise in males from 3.2% [3] to 11.2% [4] within around 7 years. A parallel trend in females is observed with an increase from 2.7% [3] to 8.8% [4] for the same period.

The genomic events accounting for loss/gain of chromosomes, loss of heterozygosity, uniparental disomy etc. are well known to have a strong correlation with CRC and precipitation of gene copy number changes [5], [6], [7]. At the functional level, several genes scheme the onset, progression and metastasis of CRC [8]. Like other cancers; the degree of copy number aberrations correlate with the incidence and severity of CRC as well as the prognosis and disease relapse [9], [10]. Areas of copy number gains and losses may affect genes critical in cancer development and reveal predictive markers that are determinant of response/resistance to treatment [2], [11]. As we appreciate the heterogeneity of these events in patient specific manner; assessment of copy number profiles can be used in personalizing treatment options to patients and in streamlining pre-treatment planning [5].

Several studies explored ethno-geographical aspects of CRC tumors and revealed putative population-specific patterns to chromosome aberrations, therefore, implying ethnical and regional basis to CRC [2], [12], [13], [14], [15]. The rapid increase in the incidence of CRC in Saudi Arabia impels to exploit advanced genomic techniques to study tumor-associated features and impart useful insights to the national disease control strategies. Although, a relevant study examining runs of homozygosity (ROH) eliminated common ancestry as a risk factor predisposing to CRC in the local population [13], a finding based on the evidence from assessment of a significantly larger and out bred population [15], yet a comprehensive characterization of CRC in the local population is still lacking. The involvement in CRC of events, such as LOH, UPD and CNAs remain largely unexplored in an integrated form.

Array comparative genomics hybridization (aCGH) reveals regions of molecular significance to cancer etiology, prognosis and remission with exemplar clarity but it is impossible to detect copy-neutral consequences such as UPDs [16] and it is hard to measure focal chromosomal events.

A recent addition to cytogenetics is the Cytoscan HD (Affymetrix) arrays through which integration and investigation of various insights to CRC is possible. The combination of single nucleotide polymorphism (SNP) based and non-polymorphic probes in this array design allow uncovering copy neutral events as well as other genomic abnormalities. It is possible to gain genome-wide insights into CNV, LOH and UPDs and bypass the inherent shortcomings of conventional aCGH [17], [18]. The ability to analyze data for different types of chromosomal events generated from a single platform allows better accuracy.

Herein, we investigated the chromosomal aberrations that could be associated with onset of CRC. By comparing tumor-normal tissue pairs in a patient-wise manner, we stress on reducing heterogeneity, eliminating cross-subject generalizations and focusing on individual subject-specific events that differentiate tumor cells from their healthy counterparts. To detect the regions of chromosomal copy number breakpoints and assess the potential of genes within them to be driving cancer we implemented two approaches: Circular Binary Segmentation (CBS) [19] and Genomic Identification of Significant Targets in Cancer (GISTIC) [20].

To the best of our knowledge, this study is the first attempt to comprehensively explore CRC associated chromosomal abnormalities in a local cohort using Cytoscan HD array yielding very high resolution captures for downstream computational analyses. We report our findings on functionality of genes affected by chromosomal anomalies including copy number gains and losses, LOH and UPD as well as transcription factor binding sites falling within these regions.

## Materials and Methods

### Ethics Statement

The study is approved by the ethics committee and Institutional Review Board (IRB) of King Abdullah International medical Research Center after due review process of the ethical aspects of the proposal. The necessary procedural and ethical consent forms were signed by each patient prior to sample collection.

### Sample Collection

Thirty biopsies were acquired from 15 Saudi patients (six males and nine females) presenting for preliminary CRC diagnosis. All cases were collected regardless of surgical stage or histologic grade. Each Hematoxylin and Eosin (H & E) stained case was reviewed by a board-certified pathologist to confirm the specimen's histological consistency with colon adenocarcinoma and that adjacent normal specimen contained no tumor cells. The sections were required to contain >60% tumor cell nuclei for inclusion in the study. The cohort consisted of patients who have not had undergone any known CRC-related clinical intervention prior to the time of biopsies acquisition.

### Sample Processing & DNA Extraction

Paired sample of tumor and adjacent normal mucosa taken from >2 cm apart was collected. Each tumor specimen weighed between 10–30 mg. The biopsy tissue was stored in RNAlater (Ambion) at 4°C for 24 hrs; followed by freezing and further storage at −20°C. CRC - positive sample pairs were then selected for DNA extraction by NucleoSpin Trio Kit (Macherey-Nagel, Germany). Quality and quantity checks were carried out by Nanodrop (Thermo Fischer Scientific).

#### Data Generation using Cytogenetics Array

Cytoscan HD arrays along with complete kit were acquired from Affymetrix (Affymetrix Inc., USA). Recommended DNA amplification kit was obtained from Clontech (Clontech Laboratories Inc., USA). The supplier's protocol was followed for amplification, hybridization, washing and staining steps. The arrays were scanned using 7000G scanner from Affymetrix. The data discussed in this publication have been deposited in NCBI's Gene Expression Omnibus and are accessible through GEO Series accession number GSE 47204.

### Data Analysis

We followed a case-control analysis strategy where the subject served as the donor of both control and tumor tissue. The tumor-normal comparisons were thus carried out between homogenous samples.

#### CNV, LOH and UPD Analysis

Nexus Copy Number 6.0 (Biodiscovery, Inc., CA, USA) was used to assess genome wide copy number frequencies for the 15 patients. Further, Aroma.affymetrix [19] – another CBS implementation as part of BioConductor's DNACopy library and the associated TumorBoost algorithm (which normalize allele specific copy numbers for tumor samples with paired normals) were also used to identify genomic events. We obtained conforming results from both implementations.

The combination of Cytoscan HD's high resolution and in depth analysis by Nexus Copy Number allows us to capture even the smallest genomic events. The Frequency threshold parameters used for the analysis are 0.2 for gain, 0.6 for high gain, −0.2 for slight loss and −1.0 for big loss. A minimum cutoff of 500 kb was used for detecting these events. Information regarding transcription factor binding sites was obtained from Open Regulatory Annotation Database (ORegAnno, www.oreganno.org) [21]. miRNA target analysis was carried out using microRNA integration system for Target Gene prediction (MIRSYSTEM) software version 20130328 available at http://mirsystem.cgm.ntu.edu.tw/

#### GISTIC analysis

Combining GISTIC (Genomic Identification of Significant Targets in Cancer) scores ranking and peaks in copy numbers of genomic regions we identified the genes according to the annotation of the human genome assembly GRCh37/hg19.

Through Nexus Copy Number we carried out GISTIC analysis. G-scores relay the significance of genes to drive cancer by weighing regions of aberration against the likelihood for random occurrence [20]. The G-scores for regions detected by CBS were examined. We labeled as significant any region of a score above 2.

#### Functional Pathways and Network Analysis

The functional and biochemical analysis for a set of 144 genes extracted from the affected chromosomal regions was done using Ingenuity Pathway Analysis-IPA (Ingenuity® Systems, www.ingenuity.com).

### Functional Analysis

Functions and diseases most significant to the dataset were identified by querying the experimentally validated Ingenuity Knowledge Base. The probability that each function/disease happened by chance alone was calculated by Right-tailed Fisher's exact test with a threshold of 0.05.

### Canonical Pathway Analysis

Pathways most relevant to the dataset were identified by the IPA library of canonical pathways in the Ingenuity Knowledge Base.

The significance of association between the data set and the canonical pathway was measured in 2 ways: 1) A ratio of the number of molecules from the data set that mapped to the pathway divided by the total number of molecules that mapped to the canonical pathway. 2) The probability that chance alone explains the association between genes in the dataset and the canonical pathways was calculated through Fisher's exact test.

### Network Graphical Representation

Two lists of genes were used to create networks: the first comprising 144 genes and the second based on high GISTIC ranks and CNV range. Graphical depiction of the pathways position gene in nodes of various shapes and the biological relationship between any two nodes is an edge. All edges are supported by at least one reference from the literature, from a textbook, or from canonical information stored in the Ingenuity® Knowledge Base. Nodes shapes represent the functional class of the gene product.

### Network Generation

Each identifier in our list was mapped to its corresponding object in the Ingenuity® Knowledge Base. Based on experimental reports this analysis filters corresponding genes and relationships found in the data registry of only humans. Then only those Network Eligible Molecules were overlaid onto a global molecular network derived from information in Ingenuity Knowledge Base. Networks of Network Eligible Molecules were then algorithmically generated based on their connectivity.

## Results

Analysis of tumor-normal pairs reveals expected chromosomal copy number aberrations yet shows patient specific variations. Preliminary evidence predicts involvement of eleven previously unreported genes in colorectal cancer Non-copy-number associated events we describe include LOH and UPD which can affect regulatory elements and cancer related genes.

### Significant Chromosomal Number Aberrations

Collectively, gains predominated chromosomes 7, 8q, 12, 13, and 20q whereas losses were common in chromosomes 1, 6, 10, 14q, 17p, 18, and 21. The short arm of chromosome 4 is yet another slightly lost region, the aberrations there and those in chromosome 6 and 10 were frequent in the female group.

**Figure 1:**
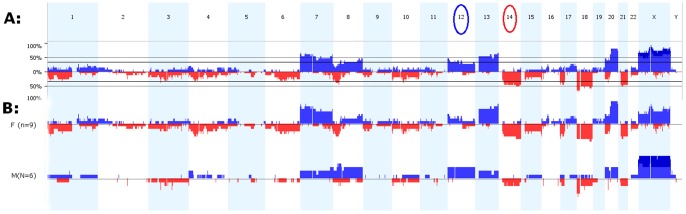
Net copy number changes. **A: Percentage of copy number gains and losses in tumor samples compared with their normal corresponding tissue for all chromosomes in fifteen study subjects.** Losses are marked in Red below the baseline whereas gains are represented in Blue bars above the baseline. Circled chromosome numbers (12 and 14) reflect events of loss and gain solely observed in our dataset. **B: Gender wise copy number changes in context of overall CNAs:** The slight loss in the short arm of chromosomes 4, throughout chromosome 6 and 10 are frequent in the female group.

A composite view of all the pair-wise comparisons is provided in (**Figure 1A**). A quick look at the gender wise clustering of the results shows more regions of losses in females (n = 9) than males (n = 6) with variable magnitude (**Figure 1B**).

Comparing the degree and frequency of losses we observe that high copy gains are more than homozygous copy losses (21 gains vs. 14 losses). The magnitude of gains therefore is higher. However we also observe that chromosomal losses occurred with more frequency than gains (285 losses vs. 273 gains). **Table 1** lists observations for each patient and gender group. Twelve subjects showed evidence of copy number gains and losses. Although losses and gains were observed in all chromosomes, unique chromosomal trends emerged for each patient and gender group. **Table 2** compares our findings against earlier published reports.

**Table 1:** Counts of copy number gains and losses per subject.

Summary of Copy Number Changes				
Subject	Gain	Chromosome	Loss	Chromosome
*Male*				
1M	6	4,8,12,X	12	2,3,10,11,16,18,20
2M	40	1,2,3,4,5,6,7,8,9,10,11,14,15,17,18,20,22,X*,Y	45	3,5,6,8,11,12,14,18,19,20
3M	11	4,8,13,20,X*	7	2,7,17,18,21
4M	39	1,4*,7,8,9,11,13,16,17,20,X*,	24	1,3,5,6,8.1,14,15,18,20,21
*Female*				
5F	13	5,9,10,12,16,17,19,20,X	10	8,14,18,X
6F	8	1,7,13,20,X	10	1,6,7,17,18,20,21
7F	28	1*,4*, 6*, 7,8,9*,11*, 14*,13,17,20,22,X*	29	1^(^ †,3,4†,7†,8†,10,14†,15,17,18,20,21,X†
8F	28	1,2,3,4,7,8,10,12,13,16,17,19,20,X*	57	1,3†,4,5,6,7,8,9,10,11,12,14,16,17,18,20,21,22,X
9F	26	2,4,5,7,8,10,11,12*,,13,17,20,21,X	28	1,2,3,4,5†,6,7,8,10,11,14,16,17,21
10F	47	1* ^,^,7*,8*,10*,13,14,16*,19* ^,^,20*,X*	50	1,2,3,4,5,7,8,9,10†,11†,12,13†,14†,15,16,17,18†,20,21,X†
11F	9	1,7,9,13,20,X	11	1,4,10,14,15,18,22
12F	18	1,3,6,7,8,9,12,13,15,20,X	2	17,X

* 21 High Copy Gain regions.

† 14 homozygous copy losses.

**Table 2:** Different CNAs in six recent studies on CRC (including our study).

	This Study	Xie et al. [35]	Jasmine et al. [5]	Ashktorab et al. [14]	Lassmann et al. [27]	Lin et al. [2]
Gain	7, 8q, 13, 20q, X, 12p *	7, 8q, 13q, 20q	7p, 8q, 13q, 20q	7, 8, 13, 20, X	7, 8q, 13q, 20q	7, 8, 13, 20
Loss	14q, 17p, 18, 1p*,4*, 8p*, 21q*	4q, 18, 21,X	4q, 5q, 8p, 17p, 18q	4,8,18	8p,15q, 17 p, 18q	4, 8, 17, 18

Underlined Text: dataset unique events.

* Slight Gain/Loss.

### Loss of Heterozygosity (LOH) and Uniparental Disomy (UPD)

**Figure 2:**
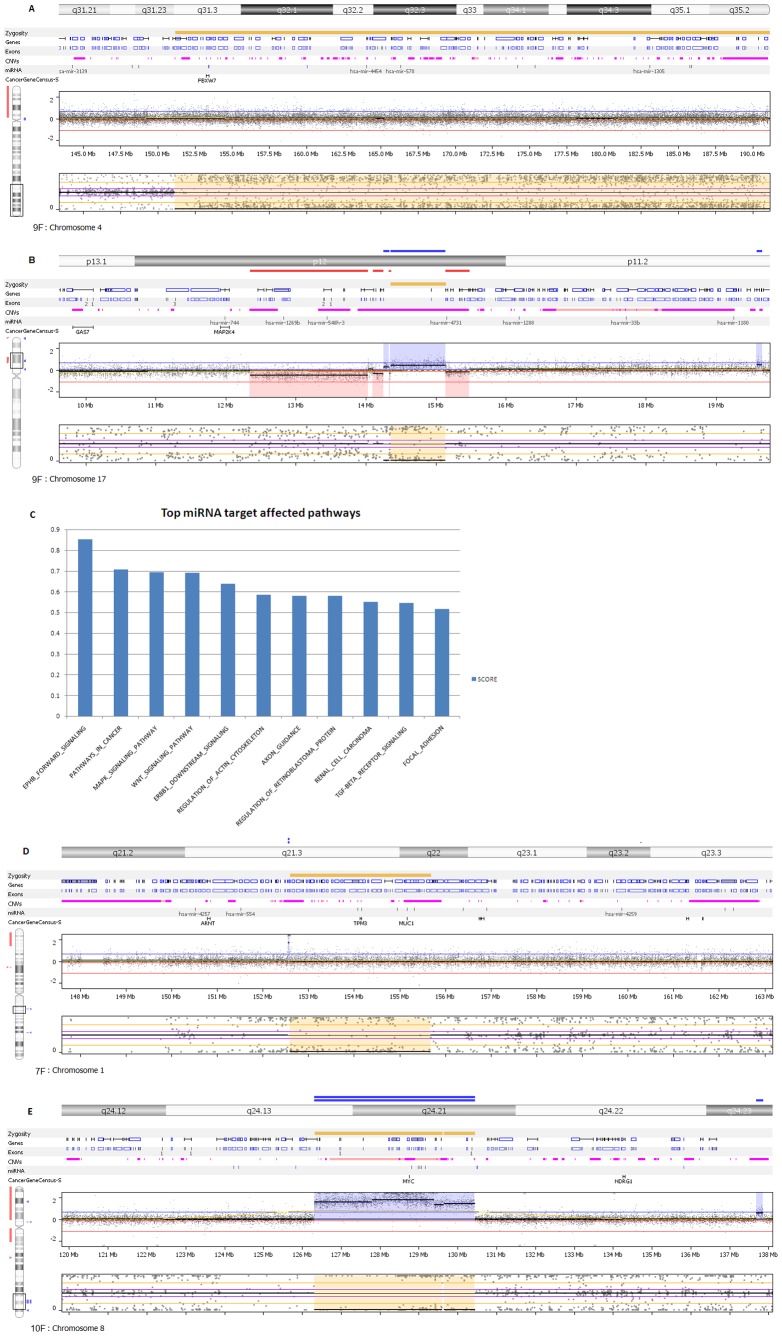
LOH and copy neutral LOH (Uniparental Disomy) events. **A:** Segmental UPD events spanning multiple microRNA regions in chromosome 4 in patient 9F. **B:** LOH event in chromosome 17 of the same sample above. LOH is associated with copy number variations. **C:** Target Prediction analysis of miRNAs exhibit the probable involvement of known signaling pathways in colorectal cancer. Y-axis represents the numerical score indicative of predictive value. **D:** TPM3 and MUC1 are affected by UPD events in patient 7F. **E:** MYC region sustain LOH events and copy gains in patient 10F.

In addition to chromosomal copy number changes, tumor-normal comparisons reveal patient specific copy neutral events. We see LOH in six cases; five of which have UPDs. Also, five of these patients have LOH/UPD events at multiple microRNA regions as depicted in **Figure 2 A and B** for chromosomes 4 and 17 in sample 9F. Target gene analysis of affected miRNAs shows their probable effect on pathways relevant to colorectal cancer. We found MAPK, WNT and TGF-β signaling pathways among the top scorers above a cutoff of 0.5 (**Figure 2C**). Complete list of all the miRNA targets and their scores are listed in **Table S1**.

Patient wise, UPD events are more frequent (total events = 15) than LOH events (total events = 7). Some patients showed extensive segmental UPDs (i.e.7F) while others (i.e.10F) showed predominating LOH; a plausible indication of different patient specific copy neutral influences on mechanisms leading to CRC.

Across patients nine cancer related genes are contained in areas pillaged by LOH and UPD. Tropomyosin 3(TPM3), Mucin 1, cell surface associated (MUC1), Thrombospondin 3(THBS3), Cbl proto-oncogene, E3 ubiquitin protein ligase B (CBLB), v-maf musculoaponeurotic fibrosarcoma oncogene homolog (avian) (MAF), v-maf musculoaponeurotic fibrosarcoma oncogene homolog (avian) (FBXW7) harbor UPDs and Cyclin D(CCND2), PCAT1 and V-myc myelocytomatosis viral oncogene homolog (avian) (MYC) have LOH events (**Figure 2 D &E**). These incidents, the length of the affected areas and the genes within are summarized in **Table 3**. The chromosomal display for the rest of the six cases is included in **Figure S1**. The functional significance of these genes was assessed by carrying out network analysis as shown in **Figure S2**.

**Table 3:** Regions of LOH and UPD.

Subject	Chr	Event	Length	Cytoband	Cancer Genes	mirna (hsa-mir-)
2M	chr3	UPD	525616	q26.1		1263
7F	chr1	UPD	3093691	q21.3–q22	TPM3, MUC1, THBS3	4258,92b, 555,5698,190b
	chr3	UPD	8846931	q13.11–q13.2	CBLB	548ab,4445
	chr8	UPD	3007553	p22		383
	chr9	UPD	1892412	p24.3		1302-11
	chr9	UPD	2967754	p11.2		
	chr16	UPD	2868239	q23.1–q23.3	MAF	4720,6504
	chrX	UPD	1071506	p22.31		
	chrX	UPD	905327	p22.31		
8F	chrX	LOH	1773583	q11.2–q12		
9F	chr20	UPD	719358	p12.3		
	chr12	LOH	4049523	p13.33–p13.31	CCND2	3649
	chr17	LOH	788743	p12		
	chr4	UPD	40000233	q31.3–q35.2	FBXW7	3140,4453,3688-1/2,4454 578,6082,548t,4276,1305,3945,4455
10F	chr20	LOH	1600308	p11.21		
	chr20	LOH	1296633	q11.22–q11.23		
	chr8	LOH	3290485	q24.13–q24.21	PCAT1, MYC	1204,1205,1206,1207,1208
	chr8	LOH	801651	q24.21		
	chr9	UPD	3485175	p11.2		
	chr15	UPD	1180310	q13.1–q13.2		
	chr17	UPD	767982	q21.31		4315-1
11F	chr20	UPD	591250	q13.33		941-1,941-3,1914,647

LOH and UPD events are seen in chromosomes of 6 subjects. Chromosomal coordinates and region length in nucleotides are listed. The genes are identified with the chromosomal map position and length of the affected area. Chromosome 20 of 9F and 10F shows significant UPD and LOH events respectively.

### Effect on Transcription Factors Binding Sites (TFBS)

**Figure 3:**
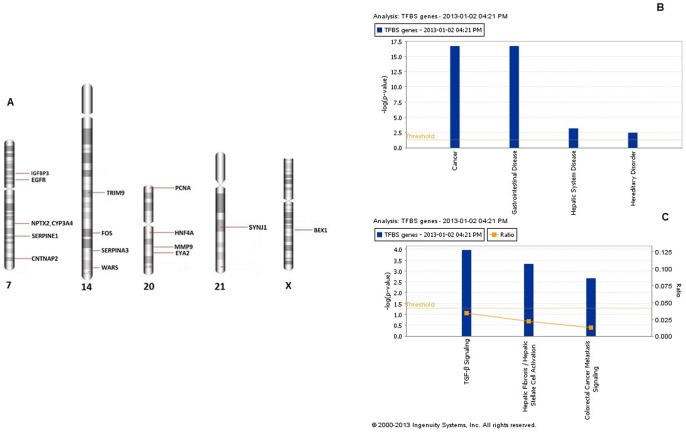
CNAs affecting transcription factor binding sites that may affect CRC related genes. **A:** TFBS in regions where the copy number changes in excess of +/−35% are marked on chromosomes 7 (gain), 14 (loss), 20 (short arm gain (males), loss (females), long arm gain (both sexes)), 21 (loss) and X (somewhat similar to chromosome 20). The marking is by the gene names corresponding to these TFBS. **B:** Association of TFBS genes with significant functions and **C:** pathways. Cancer and TGF-β signaling were statistically the most significant associations for these affected genes.

We filtered for TFBS peripheral to +/−35% copy number aberrations, which is the default cutoff value used in Nexus Copy Number 6.0. Each chromosome with affected TFBS for the respective genes is depicted in **Figure 3A**. The chromosomes 7, 14, 20, 21 and X contained CRC-related hits in addition to many others. Functional analysis of these genes supports their involvement in CRC and TGF- β signaling pathway as shown in **Figures 3B&C**. Regions of copy number gains in chromosome 7 spanned TFBS for 6 CRC related genes. TFBS for five other genes in chromosomes 14 and 21 are located in areas that demonstrate copy number losses. Some of the affected sites in chromosome 20 are located in the short arm (predominant gain in males and loss in females) while others are in the long arm for which both genders display copy number gain. A similar situation on the short arm of chromosome X is observed. **Table S2** lists all the TFBS affected on these chromosomes. **Table S4** provides details of the CNA events related to these genes.

### Functional genomics reveal 144 genes affected by copy number changes

#### Significant Genes Identification

**Figure 4:**
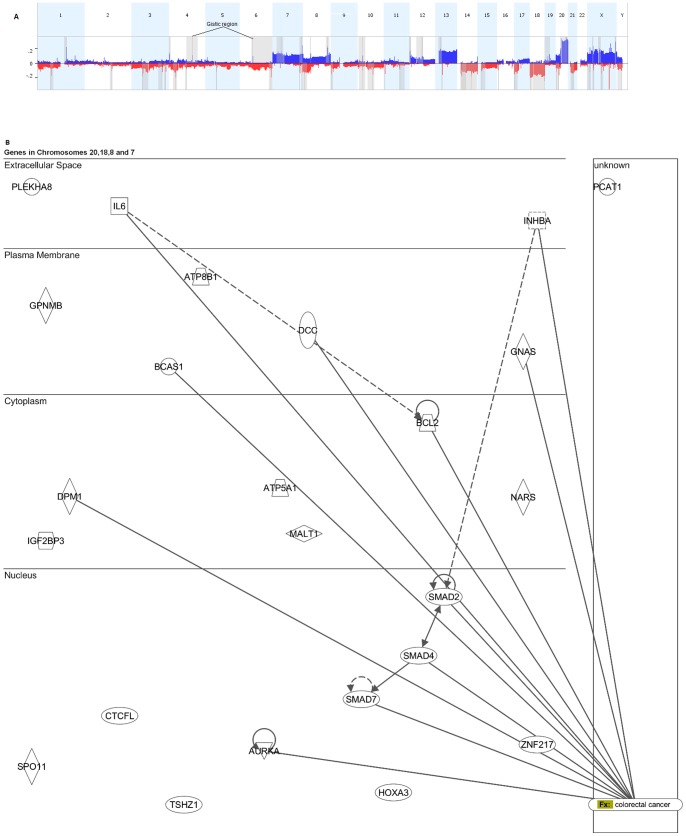
Chromosome wide GISTIC results. **A:** Amplitude of gains and losses reveal 144 genes of different GISTIC scores across all chromosomes (numbered accordingly). The spikes in the grayed out regions of enrichment correspond to GISTIC scores for genes in these regions. **B: Molecular network of genes affected by significant copy number aberrations in tumor samples.** These events were located on chromosomes 7, 8, 18 and 20. 11 of 24 genes show no association with the colorectal cancer function. This is in conformation with the GISTIC analysis except for the G-score of TSHZ1. Solid lines between nodes indicate direct molecular interaction between connected genes with respect to CRC. Functions are indicated by shapes: enzymes (diamonds), cytokines (squares), kinases (triangles), transcription factors (horizontal ovals), transmembrane receptors (vertical ovals), transporters (trapezoids), and other (circles).

144 targets of amplifications and deletions are identified by GISTIC analysis (**Figure 4A**). Ten genes in chromosome 18 are within significantly deleted regions (53% loss, G-score range 2.259–2.658). teashirt zinc finger homeobox 1(TSHZ1) has the highest G-score of 2.658 (**Table 4**). Seven genes in the gained regions of chromosome 20 (80% gain, G-score range 4.662–5.323) have high G-scores. Of these breast carcinoma amplified sequence 1(BCAS1), aurora kinase A (AURKA), GNAS complex locus (GNAS) and dolichyl-phosphate mannosyltransferase polypeptide 1, catalytic subunit (DPM1) are known in CRC (**Table 4**). At 5.32; BCAS1 has the highest G-score among all 144 genes.

**Table 4:** Chromosomes 18, 20,7 and 8 loss and gain.

Cytoband	CRC Gene	Other Gene	G-score	Function
**18q22.3**	TSHZ1		2.658	Encodes a colon cancer antigen, involved in transcriptional regulation
**18q21**	DCC		2.399	Tumor suppressor gene in colon carcinogenesis
**18q21.33**	BCL2		2.365	Regulation of apoptosis
**18q21.32**	MALT1		2.326	Tumor Suppression.
**18q21.31**		ATP8B1	2.326	Aminophospholipid Transporter
**18q21**		NARS	2.326	Asparaginyl-tRNA synthetase
**18q21.1**	SMAD2		2.259	Transcription factors and gene regulation
**18q21.1**		ATP5A1	2.259	Tumor suppressor, regulation of apoptosis
**18q21**	SMAD7		2.259	Transcription factors and gene regulation
**18q21.2**	SMAD4		2.259	Transcription factors and gene regulation
**20q13.2**	BCAS1		5.323	Oncogene, aggressive tumors.
**20q13.31**		CTCFL	4.836	Gene regulation, oncogene in ovarian cancer
**20q13.31**		SPO11	4.8360	Gene binding, ATP binding
**20q13.3**	GNAS		4.781	Oncogene
**20q13**	AURKA		4.722	Oncogene, tumor development
**20q13.1**	DPM1		4.662	Transferase activity
**20q13.2**		ZNF217	4.662	Role in breast cancer
**7p21**		PLEKHA8	2.018	Intracellular trafficking
**7p15.2**		HOXA3	2.000	Transcription factor
**7p15.3**		GPNMB	2.000	Role in metastasis
**7p15.3**		IGF2BP3	2.000	Binding protein
**7p21**	IL6		2.00	Tumor progression
**7p15**	INHBA		1.944	Cell Signaling
**8q24.21**	PCAT1		3.420	Non-protein coding prostate cancer transcript

Chromosome 18 has the steepest copy number loss (53.33%), chromosome 20 the maximum copy number gain (80%), chromosome 7 has a high gain (60%) and ranks second to chromosome 20 in terms of copy gain.

BCAS1 in chr20 has the highest G-score overall. Genes in CRC and in other cancers are identified based on the cytoband coordinates for the aberrated regions. Seven genes out of ten were involved in CRC and other three have role in various cancers in chromosome 18. G-scores indicate critical regions to drive cancer by accounting for the false discovery rate corrected significance of the frequency of gain and the average copy number for samples with gain at each probe position. TSHZ1 at the 18q22.3 shows highest G-Score value of 2.658 for observations on that chromosome with an associated copy number loss of 53.33%.

G-scores for many of the aberrations effected in other genes in chromosome 20 are as far as 3 standard deviations above the G-score mean; a range which is still higher than the immediately next uppermost G-score region (i.e. q42.21 on chromosome 8 harboring PCAT1).

PCAT1 has a gain of 46.67% in our dataset and it is the only gene in chromosome 8 with such an outstanding G-score. On chromosome 7, six genes had significant GISTIC scores of which interleukin 6 (IL6) and inhibin, β A (INHBA) are known to be associated with CRC (**Table 4**).

Ingenuity pathway analysis (IPA) infers that 12/24 genes in chromosome 7, 8, 18 and 20 are interconnected and associated with CRC. The functional implication of the high G-score genes and their network connection is portrayed in **Figure 4B**. Remarkably, eleven of the total 24 genes were not directly associated with CRC according to the IPA database.

**Table S3** lists all the 144 genes chromosomal locations and degrees of gain and loss as well as significance scores.

*Functional Analyses of GISTIC filtered Genes*

Through IPA we assessed the functional manifestation of these 144 genes. The analysis related these genes to cancer and gastrointestinal disease with high statistical confidence (−log p-value  = >55), followed by cellular differentiation, movement and other functions (**Figure S3A**).

Pathway analysis of these genes showed their involvement in hepatic fibrosis and CRC metastasis signaling. Human embryonic stem cell pluripotency and RAR signaling also had high statistical significance with a negative log p-value  = >5 (**Figure S3B**).

The top scoring network (statistical score = 40, focus molecules = 24) for this set of 144 genes comprised of the SMAD family (SMAD2, 3, 4 &7) and others which are known to be involved in cancer and a host of Gastrointestinal diseases (**Figure S3C**).

## Discussion

This study aims to address two important questions related to cytogenetics of CRC. First, Is there a common pattern of somatic chromosomal changes that fully characterize CRC? Second, what are the probable mediators of functional effect of somatic chromosomal changes in the CRC tumor cells?

To accomplish this we employed the patient tumor-normal comparison model. Our results successfully represent the unique nature of chromosomal events in each patient and conform to the observations reported by other groups yet with novel insights. We exploited the potential of a new cytogenetic microarray platform able to yield high resolution molecular data in identifying chromosomal number aberrations (gains and losses), LOH and UPD. The limitations of earlier molecular cytogenetics microarrays made it impossible to study all events in a single experiment. Our unprecedented ability to observe all these events by using a single platform minimizes variation resulting from data generation using different methods.

With an objective to understand the causal effects of acquired somatic changes that differentiates tumor cells from normal ones, we compared the cytogenetics profile of a patient's tumor with its own normal cells derived from adjacent mucosa. This approach is now being recognized as more relevant [5] as opposed to the one in which tumor samples are grouped together for comparison against a group of normal samples not necessarily from the same patient [22], [23].

While our study was in progress, a research article was published by The Cancer Genome Atlas Network [24]. It attempted providing an integrated molecular characterization of colon and rectal cancer. We used a different platform and study subjects. This provides an opportunity to explore earlier findings for translational values from a perspective of subject-wise cytogenetic events. A summarized pattern of the somatic chromosomal copy number aberrations deduced from fifteen patients was obtained using CBS.

The copy number changes we observed happen on all chromosomes representing the diversity of molecular candidates putatively involved in tumorigenesis via different mechanisms. Our results are in accordance with earlier findings that identify gains as predominant in chromosomes 7, 8q, 13, 20q and X whereas losses to be common in chromosomes 1, 8p, 17p, 18, and 21. Furthermore, with the help of the high resolution array we are able to report additional regions on 14q that carry acquired somatic CNAs. Loss of heterozygosity events at 14q12-13 and 14q32 had earlier been associated with metastatic recurrence of early-stage CRC [25] but is not linked to CNAs at the initial stage. Loss of 1p has been associated with metastatic CRC with an increased frequency and was reported in non-metastatic CRC as well [26]. Other reports of copy number losses have implicated chromosome 5 [5] and 15 [27]. These results are supported by earlier studies as summarized in Table 2.

LOH and UPD occur arbitrarily throughout the chromosomes. They impact different patients at different foci and contribute to oncogenesis, prognosis/relapse and metastasis if they compromised tumor suppressor genes. Their accidental nature warrants a within-subject exploration since each subject presents a unique case in terms of the LOH nature, frequency and affected regions. In our study, the frequency of UPD was more than twice to that of LOH. LOH events affecting tumor suppressor genes (TSGs) are believed to be a key step in CRC carcinogenesis. Though several studies attempted establishing all TSGs in the areas of LOH; the list of affected regions is growing that it is difficult to device a pattern. Rather, our observations prompt to consider these genomic events for each patient individually and emphasize the concept of personalized treatment/diagnosis approaches. In an integrated cytogenetic map by Mao et al [28] the regions 8p, 17p and 15q match with our findings in some patients where 8p22 and 15q13.1-13.2 showed UPD. Interestingly, none of these regions harbor known cancer genes.

With a diverse set of CNAs and LOH happening in a unique manner in individual patients we can answer the first question in the negative and emphasize the importance of uniqueness of a patient's genomic landscape.

We added another dimension to the effect of LOH/UPD by describing the miRNAs associated with respective regions. miRNAs may serve as better therapeutic targets than the affected regions. As evident from the miRNA target analysis the effect of LOH/UPD on the miRNAs can translate into affecting crucial pathways like WNT and TGF-β signaling. Though most of the LOH regions encompass known cancer genes including MYC, there are some areas that are still left unexplored. The relation between LOH events and their effects on miRNA profiles is largely uncharacterized especially in CRC. A recent report attempted to create an integrated picture aimed towards finding a general pattern in acute lymphoblastic leukemia [29]. A promising area for further research is to explore the connection between LOH affected cancer genes and miRNAs.

The theme of patient individuality in terms of chromosomal aberrations is reiterated by how the results for each patient yield a unique set of events affecting different genes as well as miRNAs. Given the proximity of SNP probes used in this platform the genes affected are fewer in numbers compared to earlier studies where LOH and UPDs were reported in bigger sizes. Although SNP-typing array-based CNA and LOH analyses have been reported for CRC, information for genes involved directly in such CNA and LOH is scarce [30].

Networks of the genes affected by LOH as well as UPD suggest existence of no known interactions among them and show FBXW7 and MUC1 are heavily involved in different aspects of tumorigenesis. FBXW7 is a known tumor suppressor [31] but the presence of MUC1 in UPD region is surprising as it is a well-known oncogene. Seeking connections between genes affected by LOH and UPD events we found that MYC was affected by FBXW7 and MUC affected CCND whereas PCAT is not known to interact with any other molecule in the network. The presence of MUC1 oncogene in the UPD affected region could be explained in the light of reports implicating MUC1 in suppressing cell proliferation via a complex mechanism [32]. The functional effect of LOH and UPD on these gene regions thus deserves further validation and characterization.

In order to assess the global impact of CNAs and study possible mediators of their effect, we analyzed the transcription factor binding sites that are within CNA regions. The presence of TFBS corresponding to CRC related genes in the areas of Loss/Gain indicates a probable mechanism of functional manifestation of chromosomal aberrations observed in tumor cells. Affected regions on chromosomes 7,14,20,21 and X contain TFBS related to 16 genes. These genes were found to be associated with cancer and gastrointestinal diseases. Interestingly, TGF-β signaling was a significantly affected pathway. This result resonates with functional analysis of GISTIC identified genes where the SMAD family was found to play a significant role in affecting functions and pathways. Also, miRNA target prediction analysis shows TGF- β to be significantly impacted. This analysis provides a different perspective on the plausible functional effects of compromising TFBS by CNAs and underlines the importance of noncoding regions in cancer initiation. Evidence is now being generated through efforts like ‘1000 genomes’ in linking the chromosomal changes with TFBS to better explain the functional significance of these aberrations.

While scanning regions detected by CBS we identify 91 genes known to be associated with CRC. In addition, 53 other cancer genes were incident on these regions too. Some of the genes are in areas of high gains or steep losses or are impacted by LOH and UPD. Our findings correspond with the roles these genes play in cancer as either promoters or suppressors of oncogenesis. The significance these affected regions have is conveyed in terms of GISTIC scores and reveals 24 genes in chromosomes 7, 8, 18 and 20 that could be highly critical to the transformation of normal cells to CRC tumor.

The ingenuity pathway analysis was supportive of our data in associating the 144 genes with expected functions, pathways and networks related to CRC. SMAD family of proteins constituted an important component of the most significant network in conformity with recent reports [33]. Our own analysis of TFBS affected genes implicate TGF-β signaling pathway which further stress the important causal functionality of this pathway. These studies pave the way for further validation for the functional significance of the associated genes and pathways.

Eleven genes scoring high on GISTIC analyses are ATPase, aminophospholipid transporter, class I, type 8B, member 1(ATP8B1), Asparaginyl-tRNA synthetase (NARS), ATP synthase, H+ transporting, mitochondrial F1 complex, alpha subunit 1, cardiac muscle (ATP5A1), CCCTC-binding factor (zinc finger protein)-like (CTCFL), SPO11 meiotic protein covalently bound to DSB homolog (S. cerevisiae (SPO11), Zinc finger protein 217(ZNF217), Pleckstrin homology domain containing, family A (phosphoinositide binding specific) member 8(PLEKHA8), Homeobox A3 (HOXA3), Glycoprotein (transmembrane) nmb (GPNMB), Insulin-like growth factor 2 mRNA binding protein 3 (IGF2BP3) and prostate cancer associated transcript 1 (non-protein coding (PCAT1). This suggests their novel role in the onset of CRC. GISTIC analysis is complemented by IPA which confirms their no known relation to CRC. PCAT-1 gene on chromosome 8 was found significant in our analysis and was also found to be affected by LOH event in one patient. This gene was recently implicated in prostate cancer [34] but its role in CRC is yet to be explored.

The answer to our second question thus implicates miRNA, TFBS and direct gene regions to be the mediators of functional effect of chromosomal changes. An understanding about their integrated functional pathways will be helpful in finding drug targets for CRC.

To conclude, this study emphasize on the importance of studying individual patient profile for deriving clinically relevant information and provides preliminary evidence for the role of miRNAs and transcription factor binding sites in mediating the effect of somatically acquired cytogenetic events in Colorectal cancer. Novel molecules suggested in this study need further characterization in terms of their association with colorectal cancer.

## Supporting Information

Figure S1 **Seven cases of LOH/UPD were found.** Chromosomal display of 6 patients is shown here. Patient sample are numbered and given at the bottom of each affected chromosome. M represents male whereas F represents female.
(DOCX)

Figure S2 **Functional involvement of genes in areas of UPD and LOH.** Functional networks of cancer genes impacted by UPD and LOH events with reported interactions. Pathway analysis was performed by using IPA tools' build pathway option. The solid lines correspond to direct interactions in the IPA database whereas dotted lines represent indirect interactions. PCAT 1 was not found to be connected with other molecules in the network.
(DOCX)

Figure S3 **Core analysis of 144 genes affected by significant chromosomal aberration events.** Core analysis function in IPA was employed to understand the involvement of these genes in biologically important functions, pathways and networks relevant to colorectal cancer. **A: Significant functions associated with 144 genes:** The top biological functions determined are cancer, gastrointestinal disease and cellular death and proliferation. Each of the significant biological functions is represented by blue bar. Y –axis represents-log p value as calculated by Fisher's exact test. Orange line indicates a threshold value of 0.05. **B: Significant canonical pathways affected by 144 genes:** Hepatic fibrosis, CRC metastasis were the most significant pathways as determined statistically by Fisher's exact p values represented on Y axis as −logp value. A threshold of 0.05 was used. Yellow line represents ratio of the number of molecules from the data set that map to the pathway divided by the total number of molecules that map to the canonical pathway. **C: Highest scoring network of 144 genes** shows the genes interactions. A solid line represents a direct interaction between two genes and a dotted line means there is an indirect interaction. SMAD gene family (SMAD 2, 3, 4 &7) shows most interactions in the network.
(DOCX)

Table S1 **Target analysis of miRNA affected by LOH/UPD events.**
(XLSX)

Table S2 **Transcription factor Binding Sites.**
(DOCX)

Table S3 **List of significant genes identified in CNA regions.**
(DOC)

Table S4 **CNAs associated with genes depicted in the ideogram represented in** **Figure 3A** **.**
(DOCX)

## References

[1] Jemal A, Siegel R, Ward E, Hao Y, Xu J, et al (2008) Cancer statistics, 2008. CA: A Cancer Journal for Clinicians 58: 71–96. 18287387 10.3322/CA.2007.0010

[2] Lin C-H, Lin J-K, Chang S-C, Chang Y-H, Chang H-M, et al (2011) Molecular profile and copy number analysis of sporadic colorectal cancer in Taiwan. Journal of Biomedical Science 18: 1–11. 21645411 10.1186/1423-0127-18-36 PMC3123622

[3] Al-Eid HS, Arteh SO (2004) Cancer Incidence Report 1999–2000. National Cancer Registry

[4] Haya S, Al-Eid MSM (2007) Cancer Incidence and Survival Report. Saudi Cancer Registry

[5] Jasmine F, Rahaman R, Dodsworth C, Roy S, Paul R, et al (2012) A Genome-Wide Study of Cytogenetic Changes in Colorectal Cancer Using SNP Microarrays: Opportunities for Future Personalized Treatment. PLoS ONE 7. 10.1371/journal.pone.0031968 PMC3282791 22363777

[6] Prokunina-Olsson L, Hall JL (2009) No effect of cancer-associated SNP rs6983267 in the 8q24 region on co-expression of MYC and TCF7L2 in normal colon tissue. Biomed Central 8: 1–5. 10.1186/1476-4598-8-96 PMC2777153 19895682

[7] Ho JW, Choi S-c, Lee Y-f, Hui TC, Cherny SS, et al (2011) Replication study of SNP associations for colorectal cancer in Hong Kong Chinese. British Journal of Cancer 104: 369–375. 21179028 10.1038/sj.bjc.6605977 PMC3031883

[8] Oikonomou E, Pintzas A (2006) Cancer Genetics of Sporadic Colorectal Cancer: BRAF and PI3KCA Mutations, their Impact on Signaling and Novel Targeted Therapies. Anticancer Research 26: 1077–1084. 16619509

[9] Brosens RPM, Belt EJTH, Haan JC, Buffart TE, Carvalho B, et al (2010) Deletion of chromosome 4q predicts outcome in stage II colon cancer patients. Analytical Cellular Pathology 33: 95–104. 10.3233/ACP-CLO-2010-0531 PMC4605565 20966546

[10] Sapkota Y, Ghosh S, Lai R, Coe BP, Cass CE, et al (2013) Germline DNA Copy Number Aberrations Identified as Potential Prognostic Factors for Breast Cancer Recurrence. PLoS ONE 8: e53850. 23342018 10.1371/journal.pone.0053850 PMC3547038

[11] Postma C, Koopman M, Buffart TE, Eijk PP, Carvalho B, et al (2009) DNA copy number profiles of primary tumors as predictors of response to chemotherapy in advanced colorectal cancer. Annals of Oncology 20: 1048–1056. 19150955 10.1093/annonc/mdn738

[12] Bavi PPAJ, Jeshan ZD, et al (2008) Colorectal Carcinomas from the Middle East. Molecular and tissue microarray analysis of genomic instability pathways. Saudi Medical Journal 29: 75–80. 18176677

[13] Siraj AK, Khalak HG, Sultana M, Al-Rasheed M, Bavi P, et al (2012) Colorectal cancer risk is not associated with increased levels of homozygosity in Saudi Arabia. Genetics in Medicine 10.1038/gim.2012.27 22481135

[14] Zanger U, Ashktorab H, Schäffer AA, Daremipouran M, Smoot DT, et al (2010) Distinct Genetic Alterations in Colorectal Cancer. PLoS ONE 5: e8879. 20126641 10.1371/journal.pone.0008879 PMC2811180

[15] Spain SL, Cazier JB, Houlston R, Carvajal-Carmona L, Tomlinson I (2009) Colorectal Cancer Risk Is Not Associated with Increased Levels of Homozygosity in a Population from the United Kingdom. Cancer Research 69: 7422–7429. 19723657 10.1158/0008-5472.CAN-09-0659

[16] Pinkel D, Albertson DG (2005) Array comparative genomic hybridization and its applications in cancer. Nature Genetics 37: S11–S17. 15920524 10.1038/ng1569

[17] Affymetrix Inc (2011–2012) Affymetrix CytoScan Assay User Manual.

[18] Affymetrix Inc (2011–2012) The CytoScan HD Cytogenetics Solution. P/N CL00704 Rev. 3 ed.

[19] Venkatraman ES, Olshen AB (2007) A faster circular binary segmentation algorithm for the analysis of array CGH data. Bioinformatics 23: 657–663. 17234643 10.1093/bioinformatics/btl646

[20] Beroukhim R, Getz G, Nghiemphu L, Barretina J, Hsueh T, et al (2007) Assessing the significance of chromosomal aberrations in cancer: Methodology and application to glioma. PNAS 104: 20007–20012. 18077431 10.1073/pnas.0710052104 PMC2148413

[21] Griffith OL, Montgomery SB, Bernier B, Chu B, Kasaian K, et al (2008) ORegAnno: an open-access community-driven resource for regulatory annotation. Nucleic acids research 36: D107–113. 18006570 10.1093/nar/gkm967 PMC2239002

[22] Heinrichs S, Li C, Look AT (2010) SNP rray analysis in hematologic malignancies: avoiding false discoveries. Blood 115: 4157–4161. 20304806 10.1182/blood-2009-11-203182 PMC2879098

[23] Xie T, D'Ario G, Lamb JR, Martin E, Wang K, et al (2012) A comprehensive characterization of genome-wide copy number aberrations in colorectal cancer reveals novel oncogenes and patterns of alterations. PLoS ONE 7: e42001. 22860045 10.1371/journal.pone.0042001 PMC3409212

[24] The Cancer Genome Atlas Network (2012) Comprehensive molecular characterization of human colon and rectal cancer. Nature 487: 330–337. 22810696 10.1038/nature11252 PMC3401966

[25] Al-Mulla F (2006) Metastatic recurrence of early-stage colorectal cancer is linked to loss of heterozygosity on chromosomes 4 and 14q. Journal of Clinical Pathology 59: 624–630. 16731603 10.1136/jcp.2005.033167 PMC1860407

[26] Ghadimi BM, Grade M, Monkemeyer C, Kulle B, Gaedcke J, et al (2006) Distinct chromosomal profiles in metastasizing and non-metastasizing colorectal carcinomas. Cell Oncol 28: 273–281. 17167180 10.1155/2006/529190 PMC4618002

[27] Lassmann S, Weis R, Makowiec F, Roth J, Danciu M, et al (2006) Array CGH identifies distinct DNA copy number profiles of oncogenes and tumor suppressor genes in chromosomal- and microsatellite-unstable sporadic colorectal carcinomas. Journal of Molecular Medicine 85: 293–304. 17143621 10.1007/s00109-006-0126-5

[28] Mao X, Hamoudi RA, Talbot IC, Baudis M (2006) Allele-specific loss of heterozygosity in multiple colorectal adenomas: toward an integrated molecular cytogenetic map II. Cancer genetics and cytogenetics 167: 1–14. 16682279 10.1016/j.cancergencyto.2005.08.030

[29] Ninomiya S, Tyybäkinoja A, Borze I, Räty R, Saarinen-Pihkala UM, et al (2012) Integrated Analysis of Gene Copy Number, Copy Neutral LOH, and microRNA Profiles in Adult Acute Lymphoblastic Leukemia. Cytogenetic and Genome Research 136: 246–255. 22456238 10.1159/000337297

[30] Kurashina K, Yamashita Y, Ueno T, Koinuma K, Ohashi J, et al (2008) Chromosome copy number analysis in screening for prognosis-related genomic regions in colorectal carcinoma. Cancer science 99: 1835–1840. 18564138 10.1111/j.1349-7006.2008.00881.x PMC11158266

[31] Akhoondi S, Sun D, von der Lehr N, Apostolidou S, Klotz K, et al (2007) FBXW7/hCDC4 is a general tumor suppressor in human cancer. Cancer Research 67: 9006–9012. 17909001 10.1158/0008-5472.CAN-07-1320

[32] Niv Y (2008) MUC1 and colorectal cancer pathophysiology considerations. World Journal of Gastroenterology 14: 2139. 18407586 10.3748/wjg.14.2139 PMC2703837

[33] Fleming N, Jorissen RN, Mouradov D, Christie M, Sakthianandeswaren A, et al (2012) SMAD2, SMAD3 and SMAD4 mutations in colorectal cancer. Cancer Research 10.1158/0008-5472.CAN-12-2706 23139211

[34] Prensner JR, Iyer MK, Balbin OA, Dhanasekaran SM, Cao Q, et al (2011) Transcriptome sequencing across a prostate cancer cohort identifies PCAT-1, an unannotated lincRNA implicated in disease progression. Nature Biotechnology 29: 742–749. 10.1038/nbt.1914 PMC3152676 21804560

[35] Corvalan AH, Xie T, d' Ario G, Lamb JR, Martin E, et al (2012) A Comprehensive Characterization of Genome-Wide Copy Number Aberrations in Colorectal Cancer Reveals Novel Oncogenes and Patterns of Alterations. PLoS ONE 7: e42001. 22860045 10.1371/journal.pone.0042001 PMC3409212

