# Correction: Novel Genes Associated with Colorectal Cancer Are Revealed by High Resolution Cytogenetic Analysis in a Patient Specific Manner

**DOI:** 10.1371/annotation/3f97e271-6926-4766-8430-1b4a009e80c6

**Published:** 2013-12-16

**Authors:** Hisham Eldai, Sathish Periyasamy, Saeed Al Qarni, Maha Al Rodayyan, Sabeena Muhammed Mustafa, Ahmad Deeb, Ebthehal Al Sheikh, Mohammed Afzal Khan, Mishal Johani, Zeyad Yousef, Mohammad Azhar Aziz

The name of the eighth author was given incorrectly. The correct name is: Mohammed Afzal. The correct abbreviation of the eighth author's name in the Author Contributions Statement is: MA. 

