# Supplementary figures and images for: Novel Genes Associated with Colorectal Cancer Are Revealed by High Resolution Cytogenetic Analysis in a Patient Specific Manner
Source: PLoS One. 2013 Oct 30;8(10):e76251. doi: 10.1371/journal.pone.0076251 (PMC3813709; doi:10.1371/journal.pone.0076251)

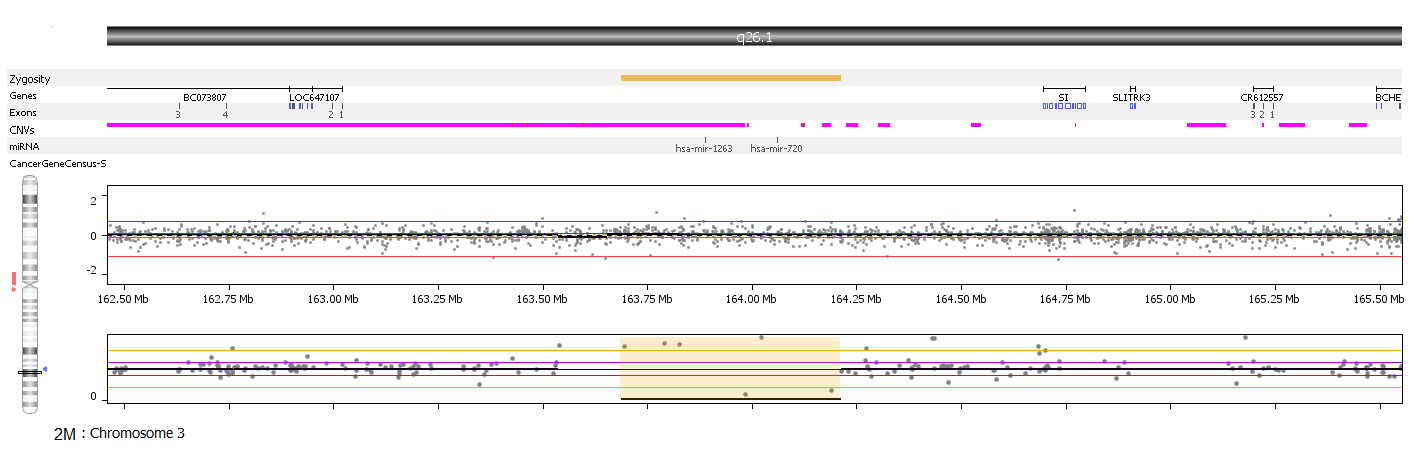

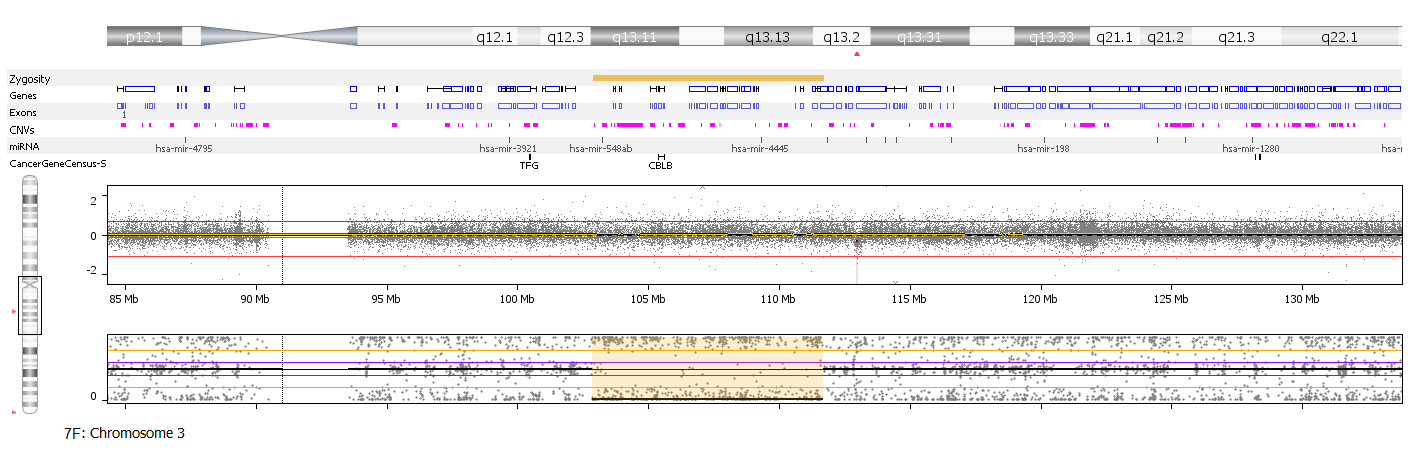

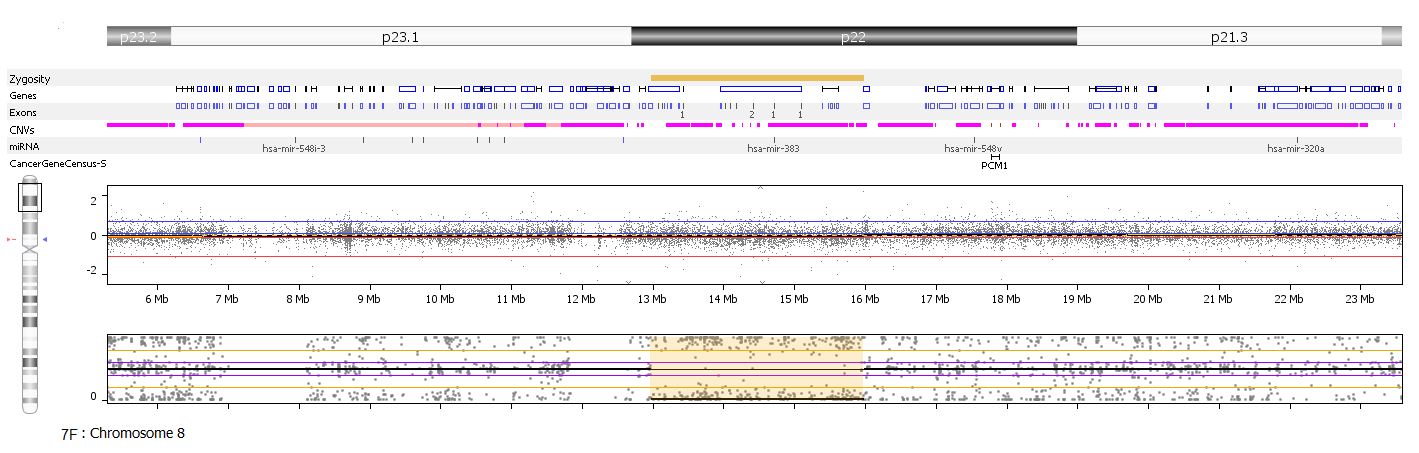

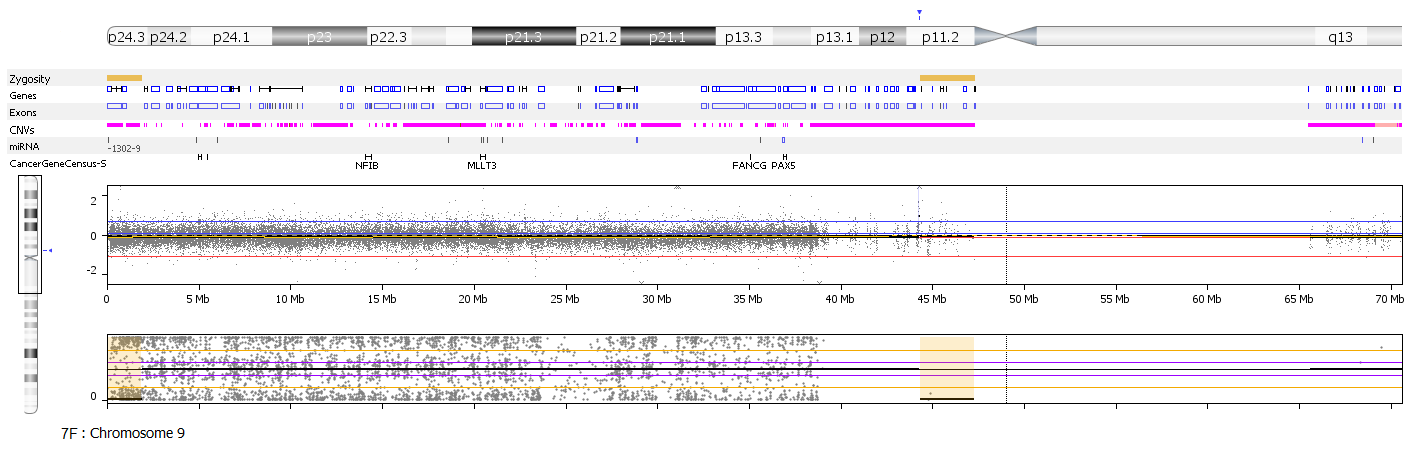

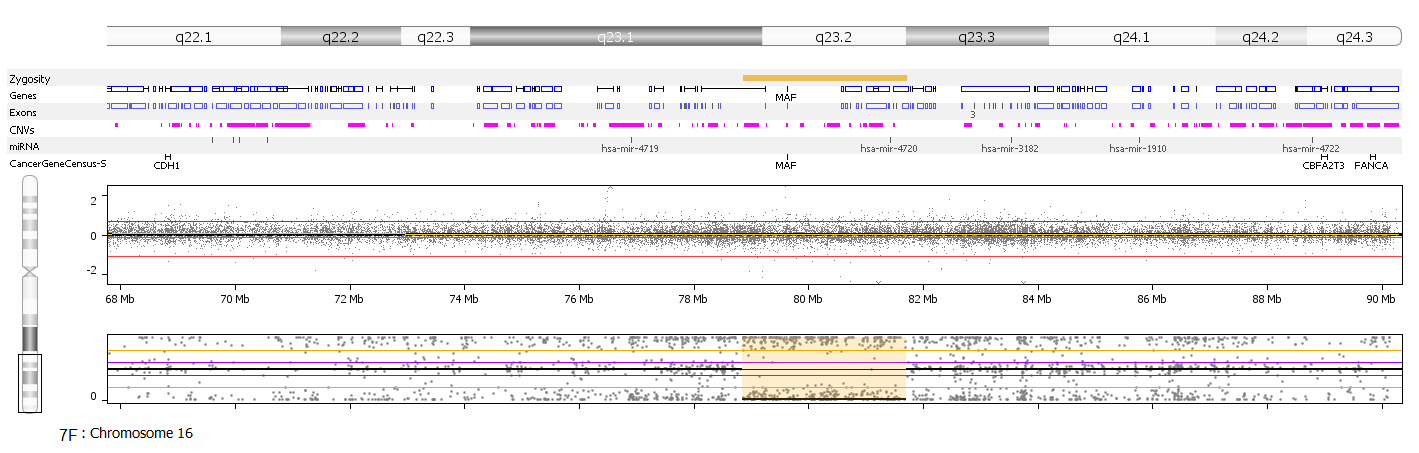

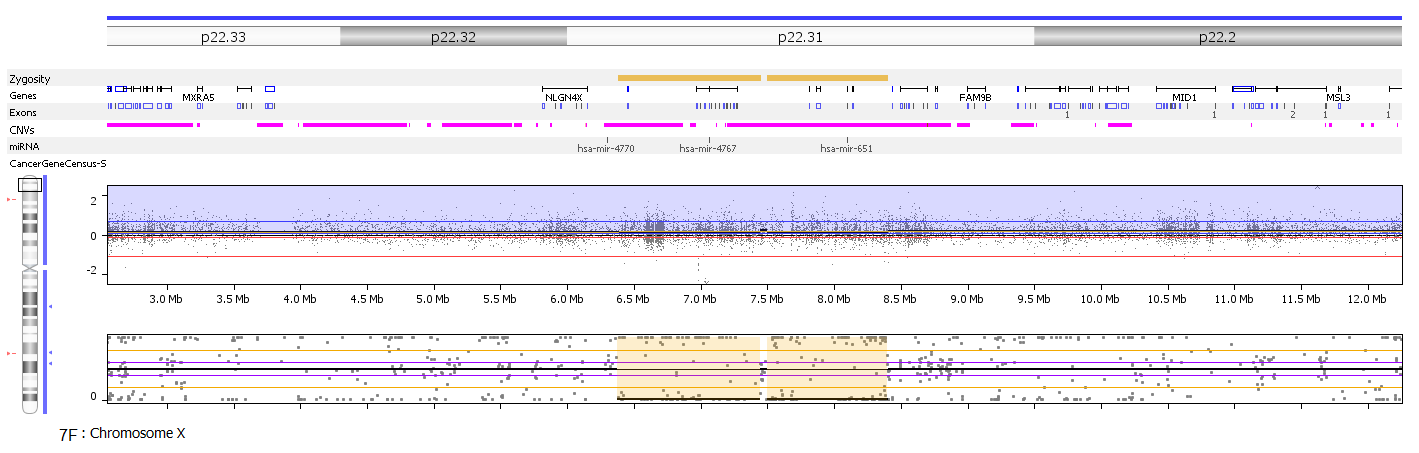

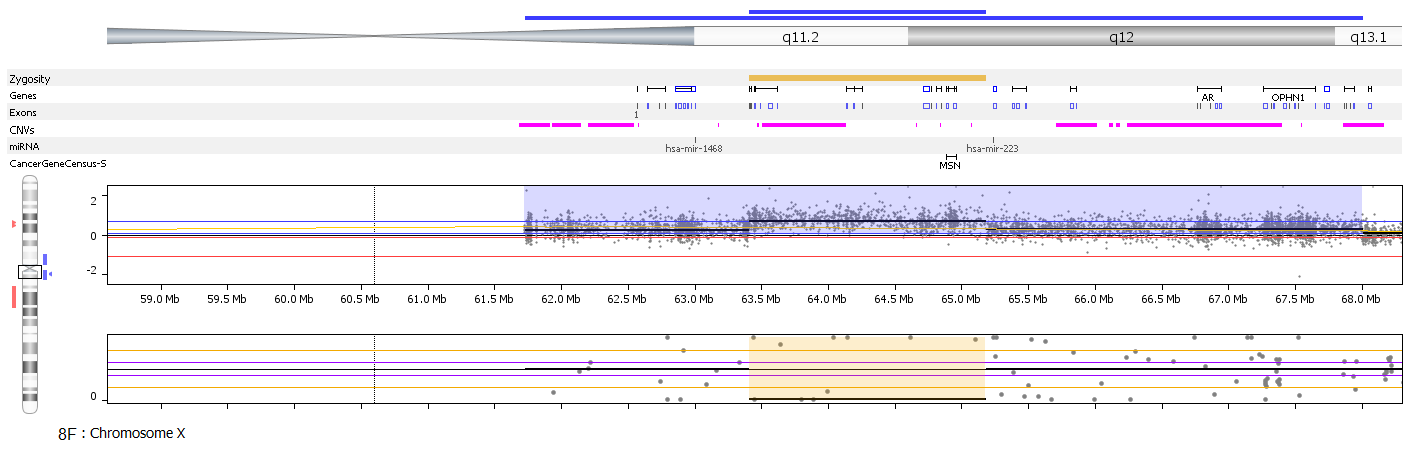

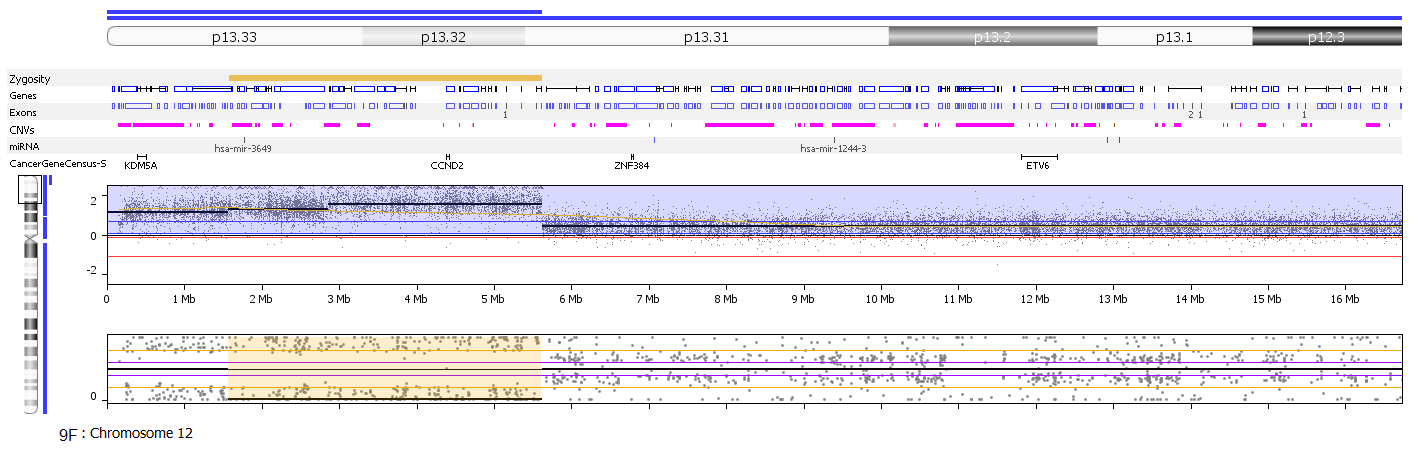

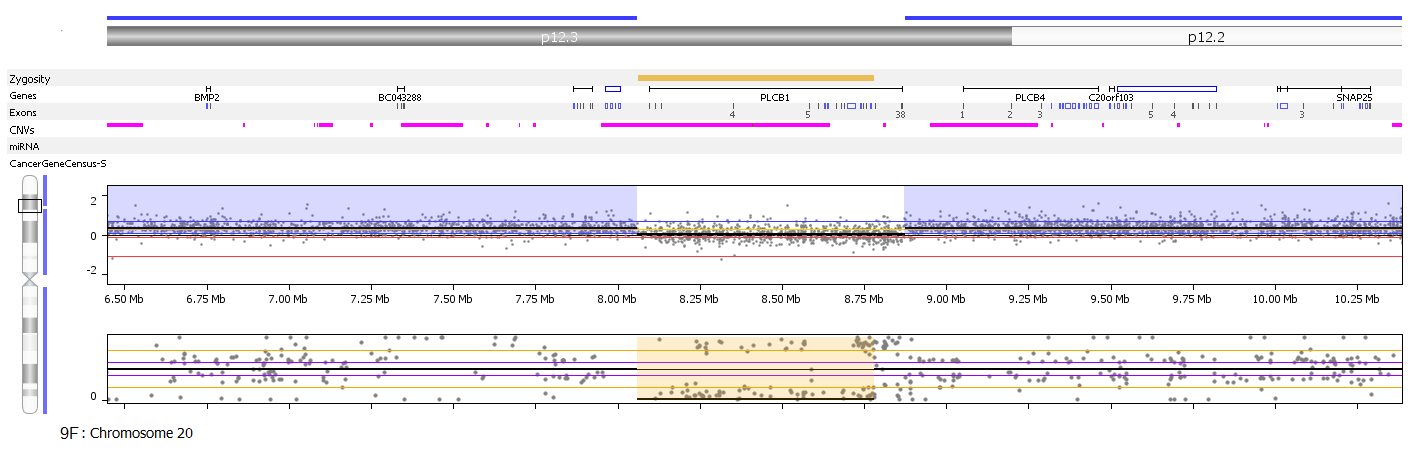

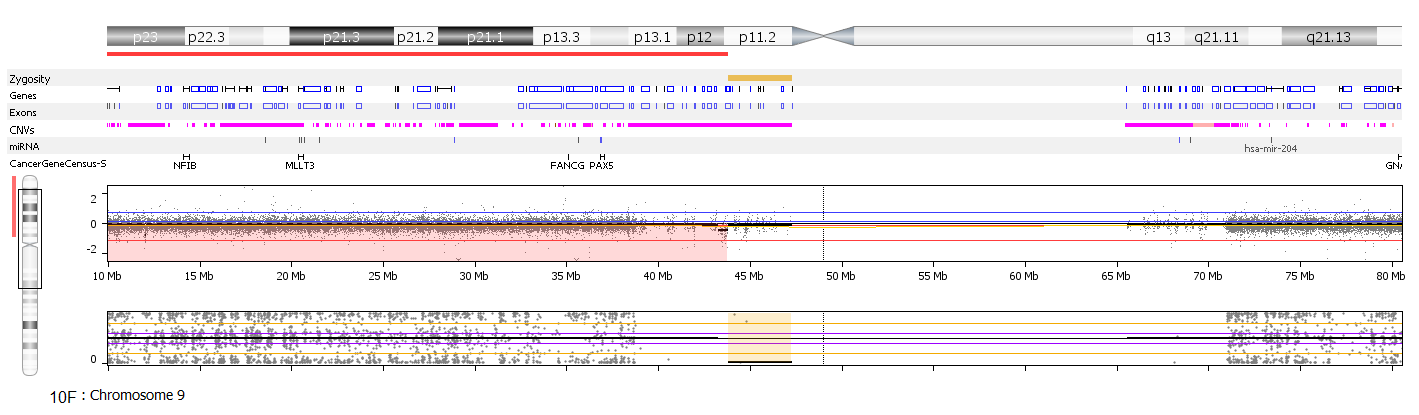

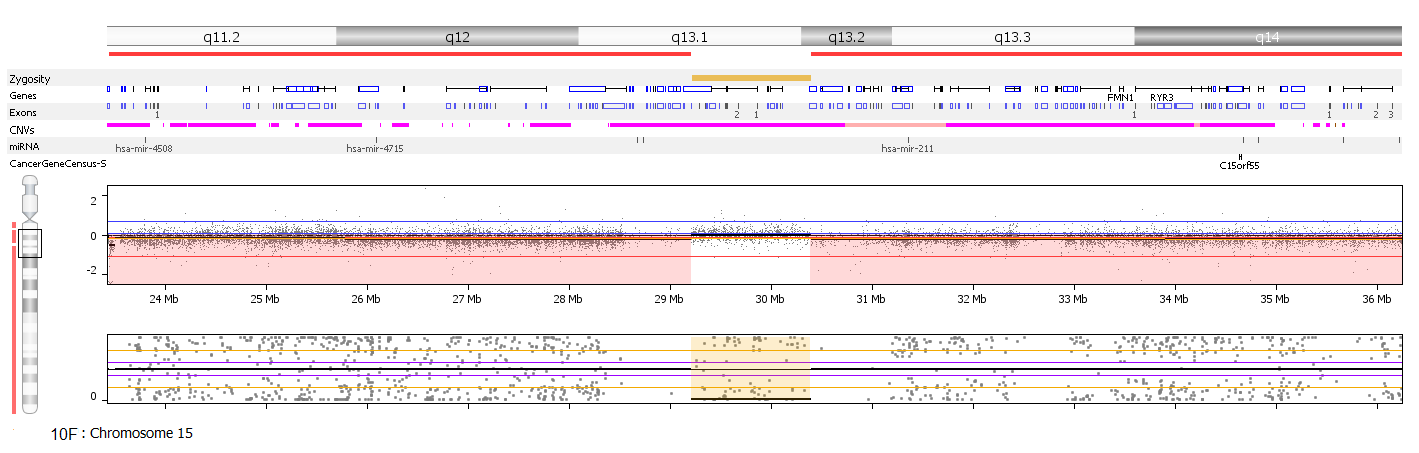

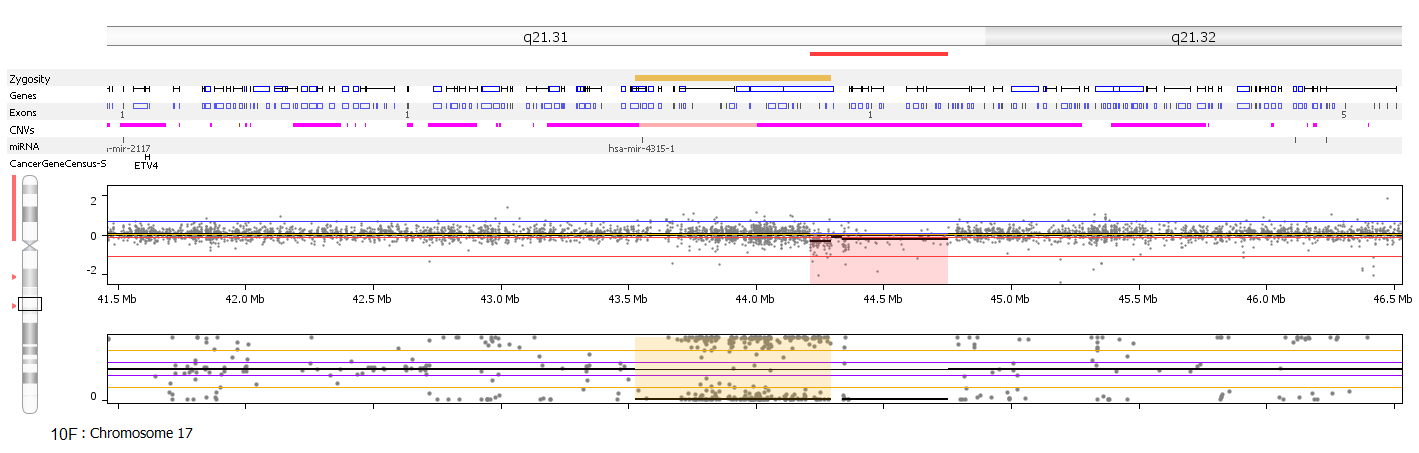

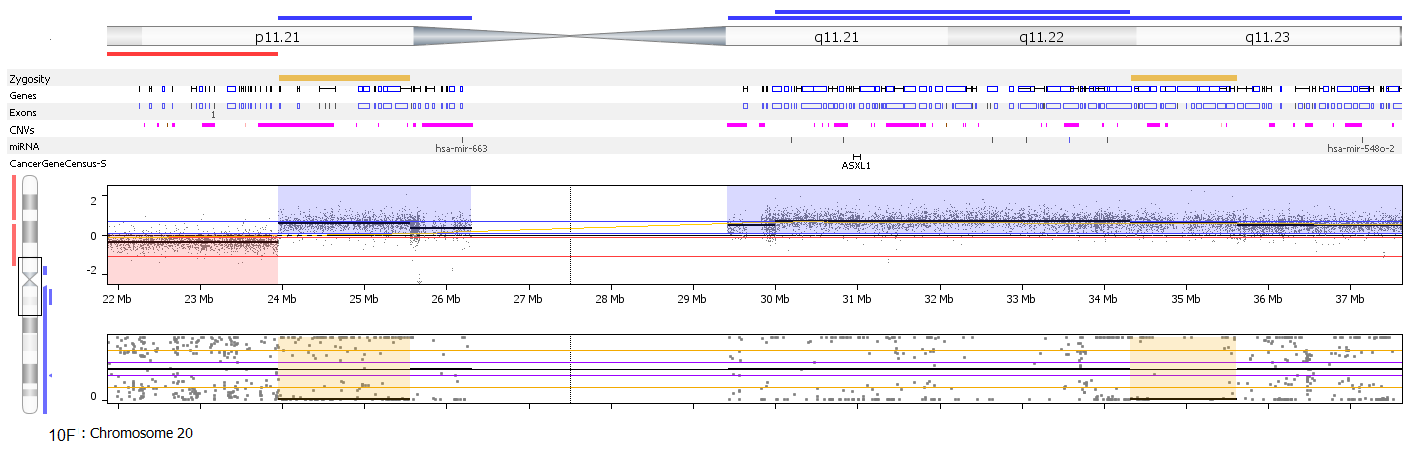

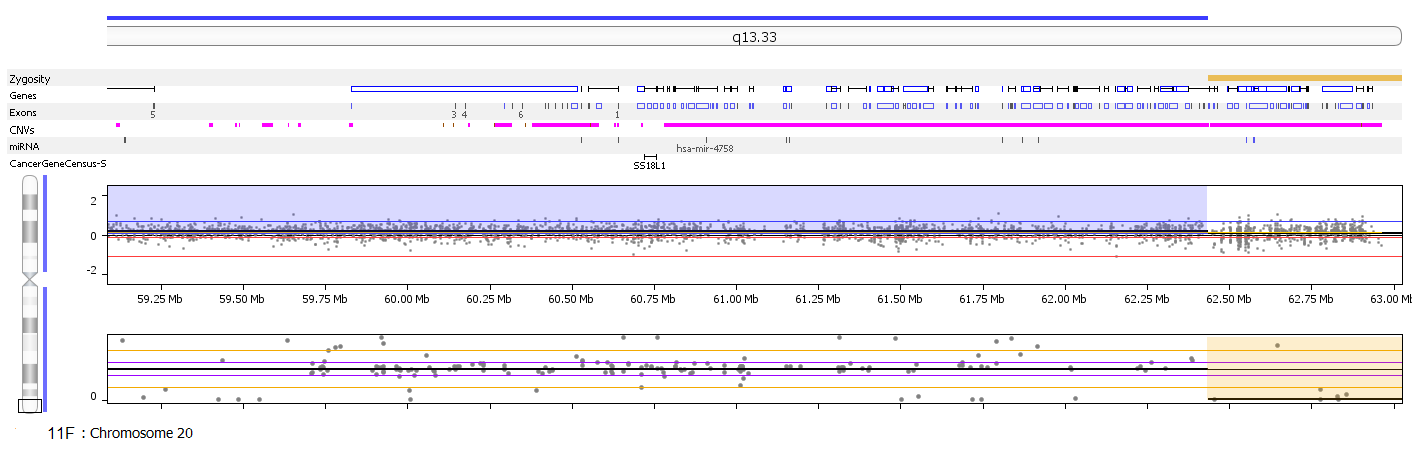

Supplement: Figure S1 — Seven cases of LOH/UPD were found. Chromosomal display of 6 patients is shown here. Patient sample are numbered and given at the bottom of each affected chromosome. M represents male whereas F represents female. (DOCX) [file pone.0076251.s001.docx]

**Figure S2: Functional involvement of genes in areas of UPD and LOH**

**
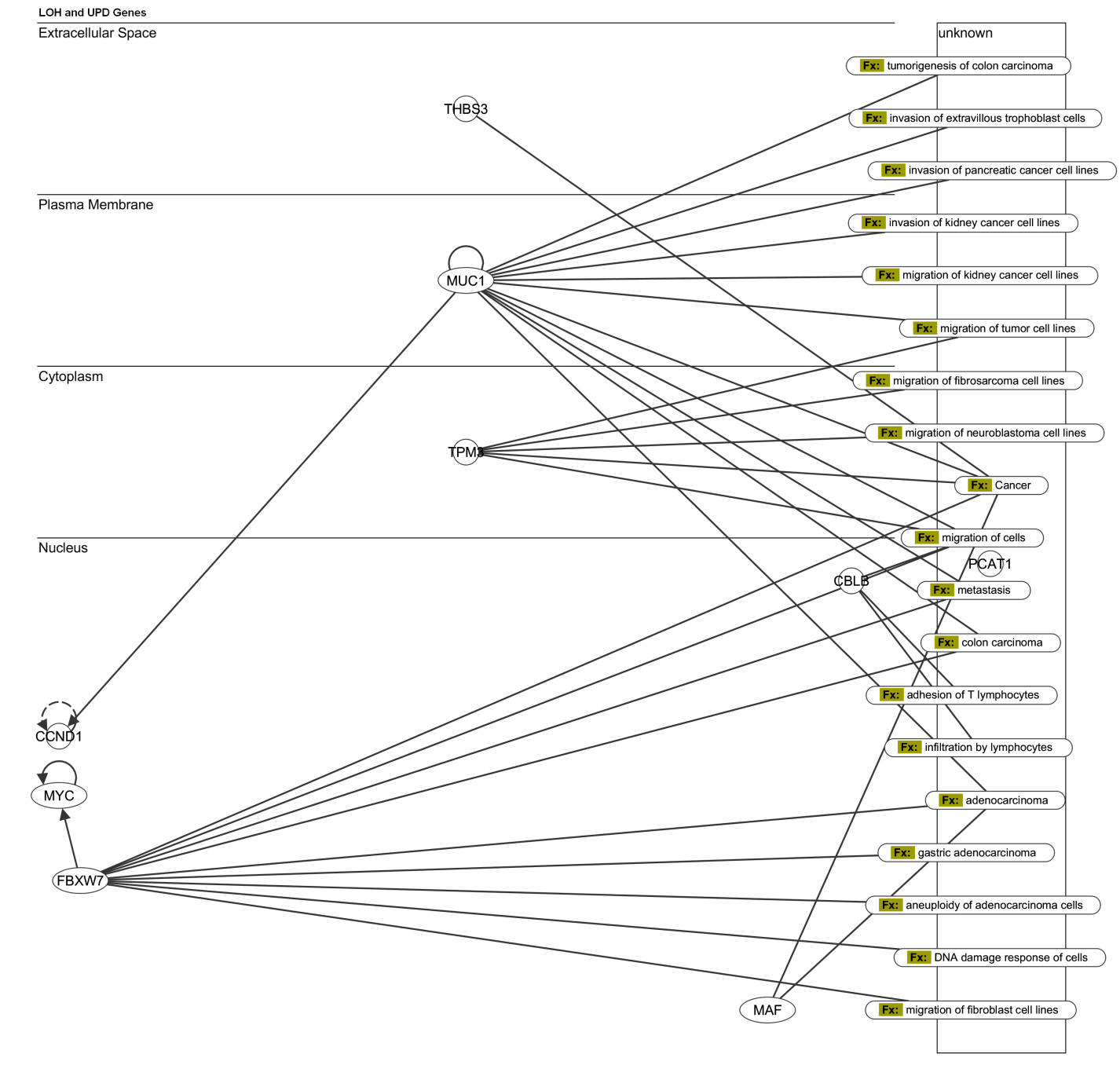
**

Supplement: Figure S2 — Functional involvement of genes in areas of UPD and LOH. Functional networks of cancer genes impacted by UPD and LOH events with reported interactions. Pathway analysis was performed by using IPA tools' build pathway option. The solid lines correspond to direct interactions in the IPA database whereas dotted lines represent indirect interactions. PCAT 1 was not found to be connected with other molecules in the network. (DOCX) [file pone.0076251.s002.docx]

**Figure S3: Core analysis of 144 genes affected by significant chromosomal aberration events**

**
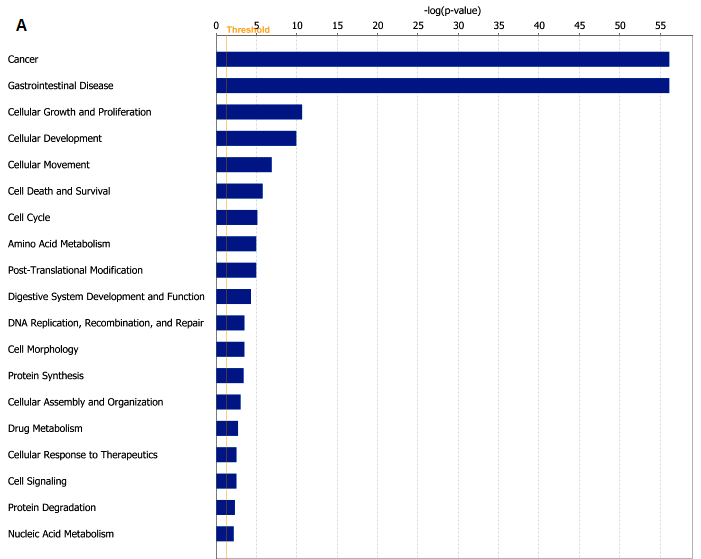
**

**
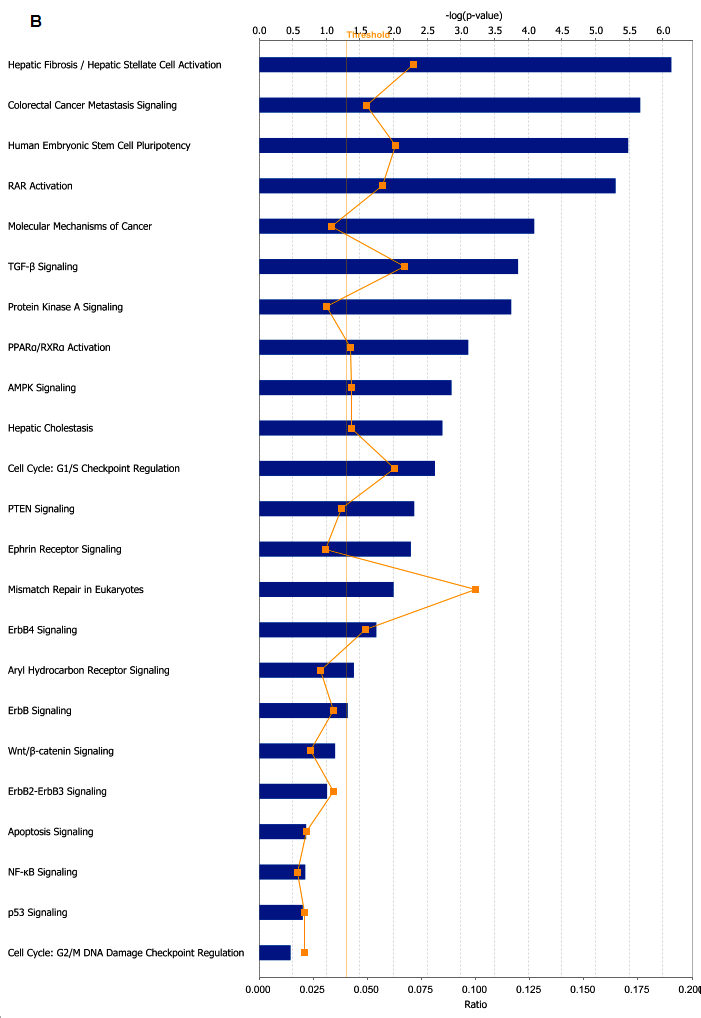
**

**
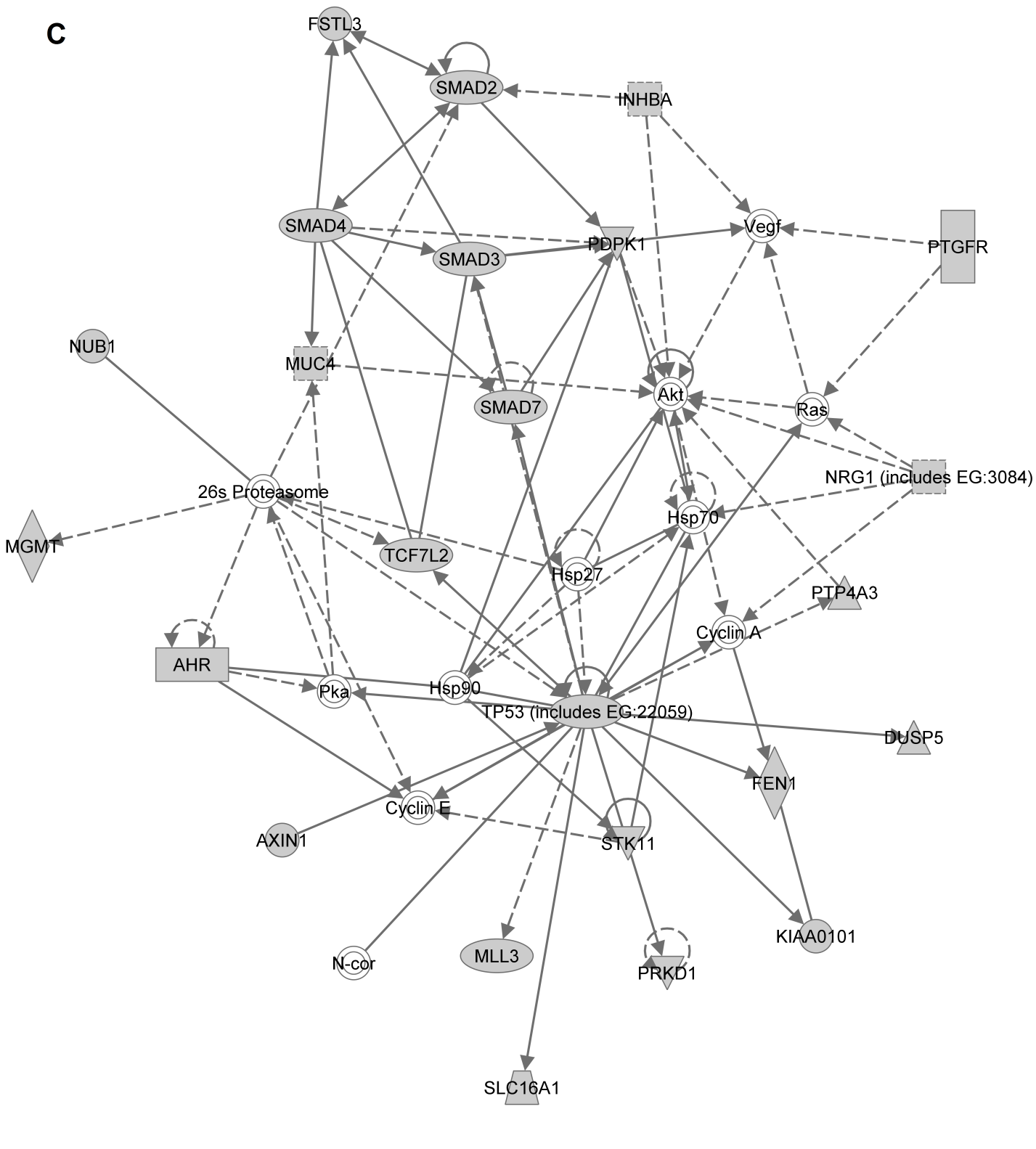
**

Supplement: Figure S3 — Core analysis of 144 genes affected by significant chromosomal aberration events. Core analysis function in IPA was employed to understand the involvement of these genes in biologically important functions, pathways and networks relevant to colorectal cancer. A: Significant functions associated with 144 genes: The top biological functions determined are cancer, gastrointestinal disease and cellular death and proliferation. Each of the significant biological functions is represented by blue bar. Y –axis represents-log p value as calculated by Fisher's exact test. Orange line indicates a threshold value of 0.05. B: Significant canonical pathways affected by 144 genes: Hepatic fibrosis, CRC metastasis were the most significant pathways as determined statistically by Fisher's exact p values represented on Y axis as −logp value. A threshold of 0.05 was used. Yellow line represents ratio of the number of molecules from the data set that map to the pathway divided by the total number of molecules that map to the canonical pathway. C: Highest scoring network of 144 genes shows the genes interactions. A solid line represents a direct interaction between two genes and a dotted line means there is an indirect interaction. SMAD gene family (SMAD 2, 3, 4 &7) shows most interactions in the network. (DOCX) [file pone.0076251.s003.docx]
