# Supplementary material for: Novel Genes Associated with Colorectal Cancer Are Revealed by High Resolution Cytogenetic Analysis in a Patient Specific Manner
Source: PLoS One. 2013 Oct 30;8(10):e76251. doi: 10.1371/journal.pone.0076251 (PMC3813709; doi:10.1371/journal.pone.0076251)
Supplement: Table S2 — Transcription factor Binding Sites. (DOCX) [file pone.0076251.s005.docx]

**Table S2: Transcription factor Binding Sites**

| Chr 7 | p22.3 - p22.2(6)( MADL1);  p15.2 - (1);  p14.1 -p12.3 (25)(AMPH, **IGFBP3**);  p12.1 (3) (DDC(2);  p11.2 - p11.1 (4)(**EGFR**(2));  q21.12 - q21.3 (3);  q21.3 -q22.1 (41)(**NPTX2**, **CYP3A4**(7), ACHE(6), MUC17, **SERPINE1**, PAI-1, VGF(3), CUTL1);  qP31.1 - q34 (70)(GRM8, SND1, CALU, **SMO**, KIAA0265, LOC442724, PLXNA4A, EXOC4, LRGUK, CNOT4,ZC3HAV1,LOC441284 (3), AGK, TAS2R4, TRBV12-4;  q35 -q36.2 (26)**CNTNAP2**, KCNH2(2),ACCN3(2), DPP6(2);  q36.2- q36.3 (14)(HTR5A(3), HLXB9, LOC392959 (2), VIPR2, PTPRN2 |
| --- | --- |
| Chr 8 | p11.23 -p11.22 (6);  q24.11q24.12 (2);  q24.13 - q24.21 (9);  q24.3 (41)(NIBP(3), FLJ43860, LOC442399(3),TSNARE1,BAI1, ARC1, CYP11B2, LOC442400, LOC338328(2), SCRT1(2), MGC70857 |
| Chr 12 | p13.33 - p13.31 (46) IQSEC3, CACNA2D4, LOC283440(3);  p13.2 - p13.1 (12);  q13.11 - q13.2 (73)(LALBA(2), NR4A1, LOC401721;  q14.3 - q15 (6) |
| Chr 13 | q12.11 - q13.3 (47)(GJB2(4), GSH1);  q13.3 - q21.31 (42) (FLJ32682, RB1(7), PCDH8);  q22.1-q31.1(8)(LOC341720, SCEL, POU4F1);  q34 (11)(IRS2, ARHGEF7, F10(2), FAM70B |
| Chr 14 | q12 - q21.1(27)(NPAS3, INSM2, TITF1(2), MIA2;  q21.3 - q22.3 (17) (**TRIM9**, CGRRF1);  q22.3 - q24.1(23)(RPL31P4, SIX6, TRMT5);  q24.1 - q24.2 (8)(SLC10A1);  q24.2 - q24.3(8)(SLC8A3(2),MAP3K9);  q24.3 (24)(LTBP2, **FOS**(7));  q31.3 - q32.31(55)(KCNK10,CHGA(2),**SERPINA3**(5),SERPINA13, LOC388010(2),**WARS**, WDR25);  q32.33(3) |
| Chr 17 | p12(6);  p11.2(7)(MAPK7) |
| Chr 18 | q22.1 - q22.3(4)(NETO1) |
| Chr 20 | p13 - p12.3(35)(SCRT2, ANGPT4,PSMF1, PDYN,CENPB,PRNT,**PCNA**, LOC388787, CHGB);  p12.1 -p11.23 (9)(PCSK2);  p11.21(2)(C20orf21, VSX1);  q11.21 - q13.33 (237)(XKR7(2),RP11-49G10.8(2),GBFA2T2(2), RALY, MMP24(2),FLJ42133,C20orf102(4),C20orf95(2),PPP1R16B,LOC339568(2),LOC440763, PTPRT(3), GDAP1L1(3),**HNF4A**, ADA(13),RIMS4(3), WFDC6, **MMP9**, SLC12A5(6), CD40, CDH22(6),LOC440764, **EYA2**, KCNB1(3), SNAI1, MC3R,C20orf66(3),PHACTR3, CDH4,, HRH3,C20orf166(2),NTSR1,LOC400852,CHRNA4, KCNQ2(3), STMN3;  q13.33(5)(MYT1) |
| Chr21 | q11.2 - q21.1(1);  q21.1 - q21.2(1);  q21.3 - q22.3(63)(**SYNJ1**, LOC440778, C21orf54, MX1(2), TFF1(2), UBASH3A(3), ADARB1, LOC441968(2), S100B(3) |
| Chr X | p11.4 (2);  p11.22 -p11.1(4);  q13.1 (4);  q13.3 - q21.1(1);  q21.32 - q22.3(9)(**BEX1**);  q22.3 - q23(3);  q24 - q25(7)(SLC25A5(6));  q27.1(1);  q28(3)(TREX2, L1CAM(2)) |
|  |  |

Number of TF binding sites for a given gene is parenthesized.

Bold CRC related.

TFBS in regions of 35% cutoff loss and gain.
