# Supplementary material for: Novel Genes Associated with Colorectal Cancer Are Revealed by High Resolution Cytogenetic Analysis in a Patient Specific Manner
Source: PLoS One. 2013 Oct 30;8(10):e76251. doi: 10.1371/journal.pone.0076251 (PMC3813709; doi:10.1371/journal.pone.0076251)
Supplement: Table S3 — List of significant genes identified in CNA regions. (DOC) [file pone.0076251.s006.doc]

**Table S3:**List of significant genes identified in CNA regions.

| **S.No.** | **Chr** | **Region in study/Band** | **Gene in CRC** | **Coordinate** | **Gene in other Cancers** | **Gain (%)** | **Loss (%)** | | **G-scores** | **HUGO ID** |
| --- | --- | --- | --- | --- | --- | --- | --- | --- | --- | --- |
| 1 | 20 | **20q13.2** | **BCAS1** | 51,993,486-52,120,711 |  | 80 | 0.00 | | **5.323** | HGNC:974 |
| 2 | 20 | 20q13 |  | 56,072,224-56,100,183 | CTCFL | 80 | 0.00 | | 4.836 | HGNC:16234 |
| 3 | 20 | 20q13.3 |  | 55,904,831-55,919,049 | SPO11 | 80 | 0.00 | | 4.836 | HGNC:11250 |
| 4 | 20 | 20q13.3 | GNAS | 57,414,795-57,486,250 |  | 80 | 0.00 | | 4.781 | HGNC:4392 |
| 5 | 20 | **20q13** | **AURKA** | 54,377,852-54,400,758 |  | 80 | 0.00 | | **4.722** | HGNC:11393 |
| 6 | 20 | **20q13.1** | **DPM1** | 48,984,812-49,008,467 |  | 80 | 0.00 | | **4.662** | HGNC:3005 |
| 7 | 20 | 20q13.2 |  | 52,183,610-52,199,707 | ZNF217 | 80 | 0.00 | | 4.662 | HGNC:13009 |
| 8 | 8 | 8q24.21 |  | 128,025,399-128,033,259 | PCAT1 | 46.67 | 6.67 | | 3.420 | HGNC:43022 |
| 9 | 18 | 18q22.3 | TSHZ1 | 72,922,731-73,001,901 |  | 0.00 | 53.33 | | 2.658 | HGNC:10669 |
| 10 | 18 | 18q21 | DCC | 49,866,542-51,062,273 |  | 6.67 | 53.33 | | 2.399 | HGNC:2701 |
| 11 | 18 | **18q21.3** | **BCL2** | 60,790,579-60,986,613 |  | 6.67 | 53.33 | | **2.365** | HGNC:990 |
| 12 | 18 | 18q21.32 | MALT1 | 56,338,618-56,417,370 |  | 0.00 | 53.33 | | 2.326 | HGNC:6819 |
| 13 | 18 | 18q2121.31 |  | 55,313,659-55,470,327 | ATP8B1 | 0.00 | 53.33 | | 2.326 | HGNC:3706 |
| 14 | 18 | 18q21 |  | 55,267,894-55,289,177 | NARS | 0.00 | 53.33 | | 2.326 | HGNC:7643 |
| 15 | 18 | 18q21 | SMAD2 | 45359466-45457515 |  | 6.67 | 53.33 | | 2.259 | HGNC:6768 |
| 16 | 18 | 18q21.1 |  | 43,664,110-43,678,287 | ATP5A1 | 6.67 | 53.33 | | 2.259 | HGNC:823 |
| 17 | 18 | 18q21 | SMAD7 | 44,700,221-44,731,079 |  | 6.67 | 53.33 | | 2.259 | HGNC:6773 |
| 18 | 18 | 18q21.2 | SMAD4 | 48,556,583-48,611,411 |  | 6.67 | 53.33 | | 2.259 | HGNC:6770 |
| 19 | 7 | 7p21 |  | 30,067,977-30,124,278 | PLEKHA8 | 60 | 0.00 | | 2.018 | HGNC:30037 |
| 20 | 7 | 7p15.2 |  | 27,145,809-27,159,214 | HOXA3 | 60 | 0.00 | | 2.000 | HGNC:5104 |
| 21 | 7 | 7p15.3 |  | 23,286,316-23,314,729 | GPNMB | 60 | 0.00 | | 2.000 | HGNC:4462 |
| 22 | 7 | 7p15.3 |  | 23,349,828-23,509,995 | IGF2BP3 | 60 | 0.00 | | 2.000 | HGNC:28868 |
| 23 | 7 | 7p21 | **IL6** | 22,733,323-22,738,145 |  | 60 | 0.00 | | **2.00** | HGNC:6018 |
| 24 | 7 | 7p15.14.1 | **INHBA** | 41,728,601-41,742,706 |  | 60 | 0.00 | | **1.944** | HGNC:6066 |
| 25 | 8 | 8p12 | **NRG1** | 32,405,728-32,622,558 |  | 26.67 | 33.33 | | **1.899** | HGNC:7997 |
| 26 | 8 | **8q24.3** | **PTP4A3** | 142,432,007-142,441,620 |  | 46.67 | 6.67 | | **1.892** | HGNC:9636 |
| 27 | 8 | **8q24.3** | **PTK2** | 141,668,481-142,011,412 |  | 46.67 | 6.67 | | **1.892** | HGNC:9611 |
| 28 | 8 | **8q24.3** | **VPS28** | 145,649,000-145,653,927 |  | 46.67 | 6.67 | | **1.892** | HGNC:18178 |
| 29 | 8 | 8q24.3 |  | 143,781,529-143,785,584 | LY6K | 46.67 | 6.67 | | 1.892 | HGNC:24225 |
| 30 | 7 | 7q36 | **INSIG1** | 155,089,486-155,101,945 |  | 60 | 6.67 | | **1.886** | HGNC:6083 |
| 31 | 17 | 17p13.1 |  | 10,395,627-10,421,859 | MYH1 | **6.67** | **40** | | 1.871 | HGNC:7567 |
| 32 | 7 | 7q36.1 |  | 151,832,010-152,133,090 | MLL3 | 53.33 | 6.67 | | 1.859 | HGNC:13726 |
| 33 | 7 | 7q36 | ABP1 | 150,549,573-150,558,379 |  | 53.33 | 6.67 | | 1.859 | HGNC:80 |
| 34 | 7 | 7q36.1 | **NUB1** | 151,038,847-151,075,548 |  | 53.33 | 6.67 | | **1.859** | HGNC:17623 |
| 35 | 17 | 17p13.1 | Tp53 | 7,577,851-7,590,863 |  | **13.33** | **40** | | 1.829 | HGNC:11998 |
| 36 | 17 | 17p13.1 | **CLDN7** | 7,103,946-7,106,519 |  | **13.33** | **40** | | **1.829** | HGNC:2049 |
| 37 | 17 | 17p13.1 |  | 149,894,981-150,071,772 | ODF4 | **13.33** | **40** | | 1.829 | HGNC:19056 |
| 38 | 7 | 7p21.1 | MACC1 | 20,174,279-20,257,013 |  | 53.33 | 0.00 | | 1.772 | HGNC:30215 |
| 39 | 7 | 7p21 |  | 17,338,276-17,385,775 | AHR | 53.33 | 0.00 | | 1.772 | HGNC:348 |
| 40 | 7 | 7p21 | **AGR3** | 16,865,555-16,888,138 |  | 53.33 | 0.00 | | **1.772** | HGNC:24167 |
| 41 | 8 | 8q24.12 | ENPP2 | 120,638,500-120,720,287 |  | 40 | 6.67 | | 1.520 | HGNC:3357 |
| 42 | 4 | 4p14 |  | 39,699,664-39,784,410 | [UBE2K](http://www.genscript.com/cgi-bin/orf/gene.pl?geneid=3093) | 0.00 | 26.67 | | 1.463 | HGNC:4914 |
| 43 | 14 | **14q11.2** | **JPH4** | 24,037,244-24,048,009 |  | 6.67 | 13.33 | | **1.437** | HGNC:20156 |
| 44 | 14 | 14q11.2 | **EFS** | 22,895,451-22,904,682 |  | 6.67 | 33.33 | | **1.437** | HGNC:16898 |
| 45 | 14 | 14q11.2 |  | 23,586,515-23,588,820 | CEBPE | 6.67 | 33.33 | | 1.437 | HGNC:1836 |
| 46 | 14 | 14q11.2 | PRKD1 | 30,045,687-30,396,899 |  | 0.00 | 46.67 | | 1.437 | HGNC:9407 |
| 47 | 5 | 5q23 |  | 121,398,890-121,414,055 | LOX | 0.00 | 20 | | 1.330 | HGNC:6664 |
| 48 | 14 | 14q11.2 |  | 19,553,365-19,584,942 | POTEG | 0.00 | 26.67 | | 1.328 | HGNC:33896 |
| 49 | 15 | 15q21.2 |  | 51500254-51630795 | CYP19A1 | **6.67** | **20** | | 1.166 | HGNC:2594 |
| 50 | 10 | **10q21** | ANK3 | 61,786,056-62,149,634 |  | **6.67** | **26.67** | | **1.157** | HGNC:494 |
| 51 | 19 | 19p13.1 |  | 15,939,757-15,946,230 | **UCA1** | 13.33 | 6.67 | | **1.155** | HGNC:37126 |
| 52 | 5 | **5q23** | **FBN2** | 127,593,601-127,873,735 |  | 0.00 | 13.33 | | **1.122** | HGNC:3604 |
| 53 | 1 | 1p31.1 |  | 78,956,728-79,006,386 | PTGFR | 6.67 | 26.67 | | 1.116 | HGNC:9600 |
| 54 | 15 | 15q21.2 | **FAM214A** | 52,873,518-52,970,820 |  | **6.67** | **20** | | **1.097** | HGNC:25609 |
| 55 | 15 | 15q21 | ADAM10 | 58,888,510-59,042,177 |  | **6.67** | **20** | | 1.097 | HGNC:188 |
| 56 | 15 | 15q21 | **RNF111** | 59,279,865-59,389,253 |  | **6.67** | **20** | | **1.097** | HGNC:17384 |
| 57 | 8 | 8p12 | **IDO1** | 39,771,328-39,786,309 |  | 33.33 | 6.67 | | **1.078** | HGNC:6059 |
| 58 | 15 | **15q15.1** |  | 45,803,334-45,848,928 | **HMGN2P46** | **6.67** | **20** | **1.017** | | HGNC:26817 |
| 59 | 15 | 15q22 | **MYO9A** | 72,118,361-72,410,422 |  | **6.67** | **20** | | **1.008** | HGNC:7608 |
| 60 | 15 | 15q22 | **KIAA0101** | 64,657,211-64,673,702 |  | **6.67** | **20** | | **0.999** | HGNC:28961 |
| 61 | 10 | 10q25 | TCF7L2 | 114,710,009-114,927,436 |  | **6.67** | **20** | | 0.961 | HGNC:11641 |
| 62 | 10 | **10q25** | **DUSP5** | 112,247,615-112,261,292 |  | **6.67** | **20** | | **0.961** | HGNC:3071 |
| 63 | 10 | 10q25 | ACSL5 | 114,135,956-114,188,138 |  | **6.67** | **20** | | 0.961 | HGNC:16526 |
| 64 | 10 | 10q26 |  | 119,806,332-119,815,736 | **CASC2** | **6.67** | **20** | | **0.906** | HGNC:22933 |
| 65 | 10 | 10q26 | **FGFR2** | 123,237,844-123,357,972 |  | 13.33 | 20 | | **0.892** | HGNC:3689 |
| 66 | 3 | 3q21 | P2RY14 | 150,929,905-150,996,230 |  | 6.67 | 13.33 | | 0.887 | HGNC:16442 |
| 67 | 3 | **3q25.1** | **HLTF** | 148,747,904-148,804,341 |  | 6.67 | 13.33 | | **0.887** | HGNC:11099 |
| 68 | 10 | 10q26 | ADAM12 | 127,702,902-128,077,127 |  | **6.67** | **20** | | 0.871 | HGNC:190 |
| 69 | 3 | 3q21 |  | 118,619,479-118,753,676 | **IGSF11** | 6.67 | 13.33 | | **0.827** | HGNC:16669 |
| 70 | 1 | 1p13.1 |  | 116,378,999-116,383,747 | [NHLH2](http://www.genscript.com/cgi-bin/orf/gene.pl?geneid=4808) | 13.33 | 20 | | 0.778 | HGNC:7818 |
| 71 | 1 | **1p13.2** | **OVGP1** | 111,956,937-111,970,399 |  | 13.33 | 20 | | **0.778** | HGNC:8524 |
| 72 | 16 | **16p13.3** | **AXIN1** | 277,441-342,465 |  | 20 | 6.67 | | **0.761** | HGNC:903 |
| 73 | 16 | **16p13.3** | **SNRNP25** | 103,829-107,669 |  | 20 | 6.67 | | **0.761** | HGNC:14161 |
| 74 | 19 | **19p13** | **FSTL3** | 676,389-683,392 |  | 13.33 | 6.67 | | **0.734** | HGNC:3973 |
| 75 | 19 | 19p1 | STK11 | 1,205,798-1,228,434 |  | 13.33 | 6.67 | | 0.734 | HGNC:11389 |
| 76 | 19 | **19p13** | **CIRBP** | 1,220,267-1,224,171 |  | 13.33 | 6.67 | | **0.734** | HGNC:1982 |
| 77 | 3 | **3q25** | **MLF1** | 158,288,953-158,324,249 |  | 6.67 | 13.33 | | **0.719** | HGNC:7125 |
| 78 | 3 | 3q21 | **RUVBL1** | 127,799,800-127,842,671 |  | 13.33 | 13.33 | | **0.717** | HGNC:10474 |
| 79 | 3 | 3q21.3 |  | 129,158,968-129,239,191 | **IFT122** | 13.33 | 13.33 | | **0.712** | HGNC:13556 |
| 80 | 6 | 6p22.1 | **HIST1H4A** | 26,021,907-26,022,278 |  | 0.00 | 13.33 | | **0.709** | HGNC:4781 |
| 81 | 3 | 3q25 |  | 156,977,532-157,221,136 | VEPH1 | 6.67 | 13.33 | | 0.707 | HGNC:25735 |
| 82 | 3 | 3q21 | MUC13 | 124,624,289-124,653,595 |  | 13.33 | 13.33 | | 0.704 | HGNC:7511 |
| 83 | 3 | 3q21 |  | 122,628,043-122,746,576 | SEMA5B | 13.33 | 13.33 | | 0.704 | HGNC:10737 |
| 84 | 6 | 6p25 |  | 4,021,569-4,065,217 | PRPF4B | 0.00 | 13.33 | | 0.694 | HGNC:17346 |
| 85 | 19 | 19p13.1 | **CHAF1A** | 4,353,660-4,394,394 |  | 13.33 | 6.67 | | **0.669** | HGNC:1910 |
| 86 | 3 | **3q29** | **MUC4** | 195,473,638-195,538,844 |  | 13.33 | 6.67 | | **0.660** | HGNC:7514 |
| 87 | 4 | 4q28 | **SLC7A11** | 139,085,248-139,163,503 |  | 13.33 | 6.67 | | **0.637** | HGNC:11059 |
| 88 | 19 | 19p13.1 |  | 16,589,875-16,607,003 | **CALR3** | 13.33 | 6.67 | | **0.619** | HGNC:20407 |
| 89 | 2 | 2q23.1 |  | 148,778,580-149,271,044 | MBD5 | 13.33 | 0.00 | | 0.598 | HGNC:20444 |
| 90 | 16 | 16p13.3 |  | 2,325,879-2,390,747 | ABCA3 | 13.33 | 6.67 | | 0.586 | HGNC:33 |
| 91 | 16 | 16p13.3 | **TRAP1** | 3,708,038-3,767,598 |  | 13.33 | 6.67 | | **0.586** | HGNC:16264 |
| 92 | 16 | 16p13.3 | **ZNF200** | 3,272,325-3,285,456 |  | 13.33 | 6.67 | | **0.586** | HGNC:12993 |
| 93 | 16 | **16p13.3** | **PDPK1** | 2,587,970-2,653,189 |  | 13.33 | 6.67 | | **0.586** | HGNC:8816 |
| 94 | 1 | **1q21.3** | **CRABP2** | 154,936,030-154,941,999 |  | **13.33** | 0.00 | | **0.565** | HGNC:2339 |
| 95 | 4 | 4q28 | **CLGN** | 141,529,057-141,568,232 |  | 13.33 | 6.67 | | **0.554** | HGNC:2060 |
| 96 | 4 | 4q32 |  | 155,702,427-155,749,965 | RBM46 | 13.33 | 6.67 | | 0.540 | HGNC:28401 |
| 97 | 3 | 3q21 | **MYLK** | 123,331,143-123,603,149 |  | 13.33 | 13.33 | | **0.535** | HGNC:7590 |
| 98 | 3 | 3q21.1 |  | 123,687,879-123,710,199 | ROPN1 | 13.33 | 13.33 | | 0.535 | HGNC:17692 |
| 99 | 3 | **3q21.1** | **ADCY5** | 123,001,143-123,167,392 |  | 13.33 | 13.33 | | **0.535** | HGNC:236 |
| 100 | 19 | 19p13.3 | **INSR** | 7,112,266-7,294,011 |  | 13.33 | 6.67 | | **0.532** | HGNC:6091 |
| 101 | 19 | 19p13.3 | **EPOR** | 11,349,475-11,356,019 |  | 13.33 | 6.67 | | **0.532** | HGNC:3416 |
| 102 | 19 | 19p13.1 |  | 15,348,301-15,391,262 | **BRD4** | 13.33 | 6.67 | | **0.532** | HGNC:13575 |
| 103 | 19 | 19p13 | ZNF442 | 12,460,185-12,476,475 |  | 13.33 | 6.67 | | 0.532 | HGNC:20877 |
| 104 | 19 | 19p13 |  | 10,400,655-10,407,454 | ICAM5 | 13.33 | 6.67 | | 0.532 | HGNC:5348 |
| 105 | 19 | **19p13.3** | **ICAM1** | 10,381,517-10,397,291 |  | 13.33 | 6.67 | | **0.532** | HGNC:5344 |
| 106 | 19 | **19p13.3** | **ANGPTL4** | 8,335,011-8,345,257 |  | 13.33 | 6.67 | | **0.532** | HGNC:16039 |
| 107 | 1 | 1q21 |  | 151,032,151-151,040,973 | **MLLT11** | **13.33** | 0.00 | | **0.529** | HGNC:16997 |
| 108 | 1 | 1q21 | **IL6R** | 154,377,669-154,441,926 |  | 13.33 | 0.00 | | **0.499** | HGNC:6019 |
| 109 | 1 | 1q21 |  | 155,165,379-155,177,772 | THBS3 | **13.33** | 0.00 | | 0.499 | HGNC:11787 |
| 110 | 19 | 19q13 |  | 48,898,132-48,948,188 | GRIN2D | 6.67 | 0.00 | | 0.492 | HGNC:4588 |
| 111 | 1 | 1q21 |  | 147,013,182-147,098,015 | **BCL9** | 13.33 | 0.00 | | **0.478** | HGNC:1008 |
| 112 | 1 | 1q42.2 |  | 233,086,370-233,114,219 | NTPCR | 20 | **13.33** | | 0.447 | HGNC:28204 |
| 113 | 1 | 1q42 | OBSCN | 228,395,861-228,566,575 |  | 20 | **13.33** | | 0.447 | HGNC:15719 |
| 114 | 1 | 1q42 |  | 229,577,044-229,644,088 | NUP133 | 20 | **13.33** | | 0.447 | HGNC:18016 |
| 115 | 1 | 1q42 | **EXO1** | 240,078,158-240,119,671 |  | **13.33** | 0.00 | | **0.405** | HGNC:3511 |
| 116 | 11 | 11q12 |  | 61,281,188-61,348,344 | SYT7 | 6.67 | 13.33 | | 0.402 | HGNC:11514 |
| 117 | 11 | **11q12** | **FEN1** | 61,560,109-61,564,714 |  | 6.67 | 13.33 | | **0.402** | HGNC:3650 |
| 118 | 16 | 16p13.3 |  | 11,913,692-11,922,689 | BCAR4 | **6.67** | **6.67** | | 0.398 | HGNC:22170 |
| 119 | 16 | 16p13.2 |  | 11,374,693-11,375,192 | PRM1 | **6.67** | **6.67** | | 0.398 | HGNC:9447 |
| 120 | 3 | **3q28** | **FGF12** | 191,857,182-192,126,838 |  | 6.67 | 6.67 | | **0.394** | HGNC:3668 |
| 121 | 19 | 19p13.1 | **LTBP4** | 41,103,141-41,135,725 |  | 6.67 | 0.00 | | **0.372** | HGNC:6717 |
| 122 | 10 | **10q26.2** | **ECHS1** | 135,025,980-135,036,898 |  | **6.67** | **20** | | **0.358** | HGNC:3151 |
| 123 | 10 | **10q26** | **MGMT** | 131,155,456-131,455,358 |  | **6.67** | **20** | | **0.358** | HGNC:7059 |
| 124 | 10 | **10q26** | **GLRX3** | 131,934,639-131,977,932 |  | **6.67** | **20** | | **0.358** | HGNC:15987 |
| 125 | 3 | **3q25** | **PTX3** | 157,154,580-157,161,417 |  | 6.67 | 13.33 | | **0.346** | HGNC:9692 |
| 126 | 1 | 1q21 | CRP | 157,948,703-157,951,003 |  | **6.67** | **6.67** | | 0.330 | HGNC:2367 |
| 127 | 16 | **16p13.2** | GRIN2A | 9,847,265-10,276,263 |  | **6.67** | **6.67** | | 0.310 | HGNC:4585 |
| 128 | 16 | 16p13.3 |  | 11,369,493-11,370,337 | PRM2 | **6.67** | **6.67** | | 0.310 | HGNC:9448 |
| 129 | 16 | **16p13.3** | **MMP25** | 3,096,682-3,110,724 |  | 13.33 | 6.67 | | **0.299** | HGNC:14246 |
| 130 | 9 | 9q32 | **PTGS1** | 125,133,229-125,157,981 |  | 6.67 | 6.67 | | **0.297** | HGNC:9604 |
| 131 | 1 | 1p12 | **SLC16A1** | 113,454,470-113,498,975 |  | 13.33 | 20 | | **0.271** | HGNC:10922 |
| 132 | 1 | 1p13.3 | **GSTM1** | 110,031,965-110,037,890 |  | 13.33 | 20 | | **0.271** | HGNC:4632 |
| 133 | 1 | 1p12 |  | 118,148,604-118,171,011 | FAM46C | 13.33 | 20 | | 0.271 | HGNC:24712 |
| 134 | 11 | **11q12** | **MS4A1** | 60,223,282-60,238,225 |  | 0.00 | 13.33 | | **0.263** | HGNC:7315 |
| 135 | 10 | **10q26.3** | **HTRA1** | 124,221,041-124,274,424 |  | **6.67** | **20** | | **0.231** | HGNC:9476 |
| 136 | 1 | 1p31 |  | 67,465,015-67,520,080 | SLC35D1 | 6.67 | 26.67 | | 0.217 | HGNC:20800 |
| 137 | 6 | 6p25 |  | 2,833,734-2,842,081 | SERPINB1 | 0.00 | 13.33 | | 0.195 | HGNC:3311 |
| 138 | 6 | 6p25 | **NEDD9** | 11,183,531-11,232,915 |  | 0.00 | 13.33 | | **0.195** | HGNC:7733 |
| 139 | 2 | 2q23.1 |  | 149,894,981-150,071,772 | LYPD6B | 13.33 | 0.00 | | 0.161 | HGNC:27018 |
| 140 | 10 | **10q25.2** | **ADRA2A** | 112,836,790-112,840,662 |  | **6.67** | **20** | | **0.152** | HGNC:281 |
| 141 | 10 | **10q26** | **EMX2** | 119,291,946-119,299,047 |  | **6.67** | **20** | | **0.152** | HGNC:3341 |
| 142 | 15 | 15q22.33 | SMAD3 | 67,358,195-67,487,533 |  | **6.67** | **20** | | 0.121 | HGNC:6769 |
| 143 | 6 | 6p22.1 | HIST1H1B | 27,834,570-27,835,359 |  | 0.00 | 13.33 | | 0.101 | HGNC:4719 |
| 144 | 9 | 9q32 |  | 117,904,097-118,164,923 | DEC1 | 6.67 | 6.67 | | 0.0231 | HGNC:23658 |

Number of CRC Genes= 91

Number of Other Cancer Genes=53
