# Supplementary material for: Novel Genes Associated with Colorectal Cancer Are Revealed by High Resolution Cytogenetic Analysis in a Patient Specific Manner
Source: PLoS One. 2013 Oct 30;8(10):e76251. doi: 10.1371/journal.pone.0076251 (PMC3813709; doi:10.1371/journal.pone.0076251)
Supplement: Table S4 — CNAs associated with genes depicted in the ideogram represented in Figure 3A . (DOCX) [file pone.0076251.s007.docx]

| TFBS | Chromosome | | | CNA | |
| --- | --- | --- | --- | --- | --- |
|  | Number | Start | End | Gain | Loss |
| IGFBP3 | 7 | 45964159 | 45964173 | ~50% |  |
| EGFR | 7 | 55086871 | 55086881 | ~50% |  |
| NPTX2 | 7 | 98243109 | 98243129 | ~50% |  |
| CYP3A4 | 7 | 99393128 | 99393146 | ~50% |  |
|  |  | 99392942 | 99392960 | ~50% |  |
|  |  | 99393109 | 99393125 | ~50% |  |
|  |  | 99392918 | 99392923 | ~50% |  |
|  |  | 99392863 | 99392868 | ~50% |  |
|  |  | 99392769 | 99392774 | ~50% |  |
|  |  | 99392817 | 99392824 | ~50% |  |
| SERPINE1 | 7 | 100769821 | 100769843 | ~50% | ~10% |
| CNTNAP2 | 7 | 145813022 | 145813042 | ~50% | ~10% |
| TRIM9 | 14 | 51561734 | 51561754 |  | ~50% |
| FOS | 14 | 75745183 | 75745193 |  | ~50% |
|  |  | 75745432 | 75745453 |  | ~50% |
|  |  | 75745466 | 75745472 |  | ~50% |
| SERPINA3 | 14 | 95078603 | 95078611 |  | ~50% |
|  |  | 95078633 | 95078641 |  | ~50% |
|  |  | 95065742 | 95065754 |  | ~50% |
|  |  | 95065896 | 95065907 |  | ~50% |
|  |  | 95065514 | 95065525 |  | ~50% |
| WARS | 14 | 100841817 | 100841827 | ~10% | ~50% |
| PCNA | 20 | 5100645 | 5100657 | ~50% | ~40% |
| HNF4A | 20 | 43025269 | 43025295 | ~50% | ~20% |
| MMP9 | 20 | 44636943 | 44636952 | ~75% |  |
| EYA2 | 20 | 45644887 | 45644914 | ~75% |  |
| SYNJ1 | 21 | 34100756 | 34100776 |  | ~50% |
| BEX1 | X | 102318253 | 102318273 | ~70% |  |

**Table S4: CNAs associated with genes depicted in the ideogram represented in Figure 3A.**
